# Cancer; an induced disease of twentieth century! Induction of tolerance, increased entropy and ‘Dark Energy’: loss of biorhythms (Anabolism v. Catabolism)

**DOI:** 10.1186/s40169-018-0193-6

**Published:** 2018-07-02

**Authors:** Mahin Khatami

**Affiliations:** Inflammation, Aging and Cancer, National Cancer Institute (NCI), National Institutes of Health (NIH), Bethesda, MD USA

**Keywords:** Accidental discoveries, Aging, Autophagy, Biological circadian rhythms, Biorhythms, Cancer biology, Cancer establishment, Conjunctiva, Constituent receptors, Crabtree effect, Dark energy, Decoy receptors, Effective immunity, Entropy, Environment, Epithelial–mesenchymal, Fatigue, Fetus orderly growth, Gastrointestinal, Growth-arrest, Growth-promote, Glycolysis, HPV vaccines, Hypoxia, Hypersensitivity, Immune intolerance, Immune-privileged, Immune-responsive, Immune suppression, Induced disease, Infection, Inflammation, PI3 Kinase, IRAK-M, Longevity, Mitochondria, Mitophagy, mTOR, Ocular tissues, Oxidative stress, Pasteur effect, P53, Personalized or precision medicine, Pyruvate metabolism, Pyruvate kinases, Pyruvate-shuttle, Phosphatases, Ribophagy, Ribosome, Targeted therapy, TNF-α, TNFRs, Viruses, Virus-like particles, Virus-contaminated polio vaccines, Yin and Yang of acute inflammation

## Abstract

Maintenance of health involves a synchronized network of catabolic and anabolic signals among organs/tissues/cells that requires differential bioenergetics from mitochondria and glycolysis (biological laws or biorhythms). We defined biological circadian rhythms as Yin (tumoricidal) and Yang (tumorigenic) arms of acute inflammation (effective immunity) involving immune and non-immune systems. Role of pathogens in altering immunity and inducing diseases and cancer has been documented for over a century. However, in 1955s decision makers in cancer/medical establishment allowed public (current baby boomers) to consume million doses of virus-contaminated polio vaccines. The risk of cancer incidence and mortality sharply rose from 5% (rate of hereditary/genetic or innate disease) in 1900s, to its current scary status of 33% or 50% among women and men, respectively. Despite better hygiene, modern detection technologies and discovery of antibiotics, baby boomers and subsequent 2–3 generations are sicker than previous generations at same age. American health status ranks last among other developed nations while America invests highest amount of resources for healthcare. In this perspective we present evidence that cancer is an induced disease of twentieth century, facilitated by a great deception of cancer/medical establishment for huge corporate profits. Unlike popularized opinions that cancer is 100, 200 or 1000 diseases, we demonstrate that cancer is only one disease; the severe disturbances in biorhythms (differential bioenergetics) or loss of balance in Yin and Yang of effective immunity. Cancer projects that are promoted and funded by decision makers are reductionist approaches, wrong and unethical and resulted in loss of millions of precious lives and financial toxicity to society. Public vaccination with pathogen-specific vaccines (e.g., flu, hepatitis, HPV, meningitis, measles) weakens, not promotes, immunity. Results of irresponsible projects on cancer sciences or vaccines are increased population of drug-dependent sick society. Outcome failure rates of claimed ‘targeted’ drugs, ‘precision’ or ‘personalized’ medicine are 90% (± 5) for solid tumors. We demonstrate that aging, frequent exposures to environmental hazards, infections and pathogen-specific vaccines and ingredients are ‘antigen overload’ for immune system, skewing the Yin and Yang response profiles and leading to induction of ‘mild’, ‘moderate’ or ‘severe’ immune disorders. Induction of decoy or pattern recognition receptors (e.g., PRRs), such as IRAK-M or IL-1dRs (‘designer’ molecules) and associated genomic instability and over-expression of growth promoting factors (e.g., pyruvate kinases, mTOR and PI3Ks, histamine, PGE2, VEGF) could lead to immune tolerance, facilitating cancer cells to hijack anabolic machinery of immunity (Yang) for their increased growth requirements. Expression of constituent embryonic factors would negatively regulate differentiation of tumor cells through epithelial–mesenchymal-transition and create “dual negative feedback loop” that influence tissue metabolism under hypoxic conditions. It is further hypothesized that induction of tolerance creates ‘dark energy’ and increased entropy and temperature in cancer microenvironment allowing disorderly cancer proliferation and mitosis along with increased glucose metabolism via Crabtree and Pasteur Effects, under mitophagy and ribophagy, conditions that are toxic to host survival. Effective translational medicine into treatment requires systematic and logical studies of complex interactions of tumor cells with host environment that dictate clinical outcomes. Promoting effective immunity (biological circadian rhythms) are fundamental steps in correcting host differential bioenergetics and controlling cancer growth, preventing or delaying onset of diseases and maintaining public health. The author urges independent professionals and policy makers to take a closer look at cancer dilemma and stop the ‘scientific/medical ponzi schemes’ of a powerful group that control a drug-dependent sick society before all hopes for promoting public health evaporate.

## Introduction

*The world will not be destroyed by those who do evil, but by those who watch and do nothing.* Albert Einstein.

Maintenance of human health is the result of an amazingly harmonious, synchronized and autonomous neurophysiological status of body. Health of an adult depends on the reciprocal interactions of positive and negative biological feedback control mechanisms (biological clocks, circadian biorhythms) between and among the immune-vascular-metabolic-genomic-hormonal-neuronal (sympathetic and parasympathetic) pathways that constitute effective immunity (biological laws) for preventing diseases including cancer. As early as the nineteenth century, Paul Ehrlich using the newly invented microscope made fundamental observations that cancer cells are destroyed by immune/inflammatory cells [1]. Furthermore, over a century ago, Peyton Rous through a series of careful and pioneering studies demonstrated that filterable viruses induce cancer; thus the establishment of the field of virology [2]. Later on in 1957, Sir McFarland Burnet conceptualized the important theory of immune surveillance, the protection of body against all unwanted elements including the control of cancer growth [3]. Burnet theory was based on evaluation and integration of a wide range of available data on cellular and molecular biology, embryology and pathology [3]. In 1986, Dvorak proposed that tumors are wounds that do not heal [4]. Furthermore, the analyses of accidental discoveries that Khatami et al. established in 1980s on experimental models of ocular inflammatory diseases, are the only series of direct evidence for an association between inflammation and time-course kinetics of induction of identifiable altered phases of immune response profiles toward multistep tumorigenesis and angiogenesis (see below) [5].

These and related fundamental observations and discoveries that stood the test of time have been practically ignored and minimized or rejected by the decision makers in cancer/medical establishment.^1^ In this perspective, the author attempted to analyze, integrate data and identify relevant knowledge gaps on diverse topics of cancer sciences toward a roadmap. The goal was to briefly present evidence that cancer is an induced disease of the twentieth century, facilitated by medical/cancer establishment for huge corporate profits ever since the American public was allowed to consume virus-contaminated polio vaccines in 1955s/1960s. Weakened immunity of public has been reinforced by other pathogen-specific vaccines (e.g., Swine flu, hepatitis B or C, measles, anthrax, meningitis, HPV or even BCG) including vaccines ingredients/adjuvants (e.g., mercury, aluminum, l-histidine, recombinant DNA, embryonic serum), which are ‘antigen overload’ for the immune system to clear. In the last few decades, public has additionally been exposed to a wide range of environmental hazards [e.g., smoking, pesticides, genetically modified organisms (GMOs) and preserved foods, electronic gadgets, low level carcinogens] and other immune disruptors whose cumulative effects adversely influence immunity [5–25].^2^, ^3^, ^4^

## Accidental discoveries in 1980s: time course kinetics of inflammation-induced developmental phases of immune dysfunction toward multistep tumorigenesis and angiogenesis

In 1980s, we established experimental models of acute and chronic ocular inflammatory diseases using guinea pigs conjunctival-associated lymphoid tissues (CALTs). Systematic analyses of a series of reported data suggested the first and only evidence for direct association between inflammation and time course kinetics of developmental phases of immune dysfunction (acute, intermediate and chronic phases) in the direction of tumorigenesis and angiogenesis (‘accidental’ discoveries) [5–8, 26–41]. Briefly, in the acute phase (immediate hypersensitivity responses), early clinical and histopathological findings included strong or weak type 1 ocular reactions, tearing, activation and degranulation of mast cells (MCs), vascular hyperpermeability reactions and tissue edema. The release of histamine and prostaglandins (PGF-1α) were reported as primary and secondary mediators of immune responses in acute phase reactions. Animals with strong acute ocular reactions also demonstrated wheezing suggestive of MCs sensitization and activation in lung airways [8, 26, 30, 31]. The intermediate phase (down-regulation phenomena) responses were associated with minimum clinical responses, increased degranulated MCs, heavy infiltration of eosinophils in ocular secretions and goblet cells, induction of neovascularization and tissue atrophy [27, 31]. In the chronic phase responses, clinical appearance of tumor-like lesions in upper and lower bulbar conjunctiva, extensive angiogenesis and massive hyperplastic tissues were reported. Histopathological studies demonstrated loss of capsular integrity in lymphoid tissue, increased presence of various size lymphocytes (proliferation), lymphatic channels, necrosis and growth in epithelial tissues, activated MΦs and presence of histiocytes (activated DCs?). Antibody assays of hyperplastic tissues showed changes in local IgGs biosynthesis (IgG1/IgG2 ratios). New born guinea pigs from highly sensitized animals developed strong clinical reactions upon 1st or 2nd challenge with antigen suggesting antibody transfer and genetic predisposition in newly born animals [8, 28, 29, 31]. Mixing antigen with tumor-promoting agents shifted the induction of tumorigenesis and angiogenesis to earlier time course, compared with using antigen alone, suggesting involvement of growth promoting kinases. Presence of circulating IgE antibodies did not necessarily correlated with strong acute reactions, suggesting sensitization of local and distal MCs including the lung airways or the fetus tissues [8, 26, 31]. In 2014, further analyses and integration of original data led to the first report on interactions and synergies between host/local immune and non-immune cells (e.g., mast cells, mucus-secreting goblet cells, epithelium, B/plasma cells) and the recruitment and infiltration of activated immune cells (e.g., eosinophils, tumor associated macrophages/TAM-M2) via activation of vasculature toward tumorigenesis and angiogenesis at different stages of immune dysfunction [38].

These are the earliest and only series of systematic studies that produced evidence on the role of repeated exposures to immune disruptors (antigen) in the induction of multistep tumorigenesis and angiogenesis. These accidental discoveries on direct role of inflammation in alterations of immune dynamics toward tumorigenesis and angiogenesis deserve to be further studied, validated, confirmed and compared with other experimental models of inflammatory diseases, other tissues [e.g., lung-associated lymphoid tissues (LALTs), gut-associated lymphoid tissues (GALTs)] or other tissues with different cell compositions (e.g., liver, breast, colorectal).

As detailed elsewhere ([5–7, 38, 39], Khatami, NCI/NIH records and legal documents since 1998), author’s challenging efforts to promote the important role of inflammation in cancer research, diagnosis and therapy initially met with serious oppositions and denials by members of establishment who rejected the submitted concepts and comprehensive proposals that were extension of author’s original discoveries. However, in recent years, it seems that Khatami’s efforts awakened the entire cancer community within and outside NCI/NIH, on the important role of inflammation in cancer science. In the last 2 decades, professionals enjoy significant increased in funding on fragmented submitted ideas in the fields of OMICS (e.g., proteomics, genomics, metabolomics, lipidomics, proteo-genomics) or immunotherapy, using highly expensive specific technologies and experimental models [e.g., genetically or chemically-induced (e.g., comination of azoxymethane (AOM) and dextran sodium sulfate)] of tumors and organizing networking and symposia, with no end in sight [5–7, 38–43]. However, the outcomes of reductionist approaches on identification of hundreds of molecular entities that are used for drug development and claimed as ‘targeted’ therapy or ‘personalized’ or ‘precision’ medicine have been very disappointing, extremely costly and dangerous for patients and society. Majority of expensive projects in cancer research and therapy focus on identification of too many defective molecular species in the landscape of cancer molecular tsunami with little/no efforts to understand what initiate altered immune response profiles that lead to multistep tumorigenesis. The initial immune response alterations were suggested to be correctable, reversible or drugable [5, 7, 16, 39–43].

Ongoing debates, controversies, misinformation and confusions regarding the role of inflammation, whether it is protective in preventing cancer or it is a cause of cancer are among serious factors in failing patients when clinical trials are decided [5–8].^5^

## Categories of human diseases

To better understand the biology of cancer, we first define that, in general, all diseases are the outcomes of one or combination of interdependent biological defects that are medically/clinically categorized as the following:

1. Congenital.
2. Hereditary.
3. Neonatal.
4. Induced.

In the following sections, attempts were made to demonstrate and suggest that cancer is a symptom of accumulated violations of time-controlled biorhythms whose disorderly proliferation and mitosis are facilitated by altered mitochondrial bioenergetics, increased glucose utilization and entropy and generation of ‘dark energy’, conditions that are toxic to normal cells. Unlike the popularized notion that cancer is 100, 200 or 1000 diseases!, the author shows that cancer cells are body’s defective cells whose disorderly growth are the results of loss of natural biphasic properties of Yin (tumoricidal) and Yang (tumorigenic) of inflammation, associated with loss of differential bioenergetics (anabolic vs catabolic) in tissues. Evidence is presented that over the last six decades, decision makers in cancer establishment has gradually weakened and manipulated the autonomic biological circadian rhythms, the Yin and Yang of effective immunity, by introducing the public to various pathogen-specific vaccines and ingredients, in addition to exposures to exposures to a wide range of environmental hazards and low level carcinogens that made young and old in America sick and drug-dependent.

Analyses of related data suggest that nearly all diseases, to varying degrees, are the results of altered effective immunity or loss of biological circadian rhythms (on–off switches) that adversely influence tissue bioenergetics (mitochondria), the principal generator of energy and electricity (proton pumping) in organ systems [5, 7, 35–90].

It is also noteworthy that the special or shared biological/medical features of full-blown diseases that often determine the outcomes of nearly all age-associated chronic conditions fall into the following three major categories, as interdependent defects in immune surveillance (biorhythms) associated with varying degrees of loss of bioenergetics in affected tissues [5, 39]:

- Vascular and lymphatic channels disorders.
- Tissue necrosis.
- Tissue growth.

Manifestations of organ-specific diseases that are known as distinct pathological and clinical complications such as polyps, pre-cancer, cancer metastasis and angiogenesis, or neurodegenerative and autoimmune diseases, diabetes and cardiovascular complications, are generally the outcomes of cumulative damages in the immune-responsive tissues (e.g., epithelial, endothelial, mucus secreting goblet cells, stroma) or immune-privileged tissues (e.g., CNS, BBB, neuroretina, cornea, reproductive system) and/or insulin-dependent (e.g., muscle, adipose tissue, liver) and insulin-independent tissues (e.g., vasculature, neuronal and endothelial cells, fibroblasts) for glucose transport and metabolism [5–8].

Overview and integration of data on diverse experimental models of chronic illnesses or cancer research and clinical studies suggest that all diseases share features of one or more defects in innate (intrinsic, constituent) and/or adoptive (extrinsic or induced) pathways including cellular [e.g., genomic/chromosomal (DNA/RNA, epigenetic modifications), cell mediated or humoral immunity (CMI/HI), hormonal, metabolic, neuronal (sympathetic and parasympathetic)] activities that contribute to the complex signal communications of altered immunity and manifest in different tissues as different diseases [5–8, 34–80]. We proposed that effective immunity (synchronized biorhythms, sympathetic and parasympathetic, or Yin and Yang of acute inflammation) is responsible for protection of body against all intrinsic (e.g., defective cancerous cells, useless proteins/peptides/lipids, accumulated oxidized materials, lymphocyte-derived clonal complexes, abnormal repair mechanisms in chromosomal-histone proteins-genetics, DNA/RNA mutations, or hypo/hyper epigenetic modifications) or extrinsic elements (e.g., pathogens/microbiomes, allergen/antigens, low level carcinogens) that are perceived harmful to individual’s survival [5–8, 20, 35–39].

## Bioenergetics of effective immunity: biological circadian rhythms via biphasic-synchronized Yin and Yang of acute inflammation

As detailed elsewhere [5–8, 35–40], effective immunity follows complex and precise rules of molecular engagements and require development of differential bioenergetics for body’s defense and survival after birth. Effective immunity was defined as a highly regulated signal transductions between 2 biologically opposing arms, termed Yin (tumoricidal) and Yang (tumorigenic) that intimately engage the activities of immune-metabolic–vasculature–hormonal–neuronal (sympathetic and parasympathetic) systems.

In an acute inflammation, autonomic crosstalk between innate and adaptive immune cells and non-immune pathways has a 2-fold mission with differential energy requirements from mitochondria and cytosol as outlined below [5, 35–40].

### A. Catabolic (Dressed to Kill!), Yin (tumoricidal arm): high energy consuming events utilizing oxidative phosphorylation from mitochondria

Yin events are involved in encountering/sensing (recognizing), processing or digesting and eliminating the intrinsic or extrinsic foreign elements (e.g., allergen, pathogens/microbiota, cancerous cells, oxidized metabolites, senescent cells or lymphocyte complexes) and the injured host tissue. Yin (apoptotic, pro-inflammatory) events require burst of energy (ATP hydrolysis) from mitochondrial oxidative phosphorylation (OxPhos) for activation of innate and adoptive immune cells [e.g., MCs degranulation, MΦs (M1 phenotype), DCs, NKs or T and B/plasma cells], as well as activation of non-immune systems (vasculature, metabolism, neuronal, hormonal) for generation of required toxins, oxidants and apoptotic factors. Pathogen-specific induction of danger molecules and expression of specific pro-inflammatory mediators and receptor molecules (e.g., TLRs, vasoactive histamine, TNF, ILs, ROS, NO, complement cascades) as well as, secretory lysosomal activities, membrane fusion, exocytosis, ion fluxes and vascular hyperpermeability responses facilitate the destruction of foreign elements and injured host tissue. Yin events require genomic activation of specific tumoricidal factors (above examples) [5–8, 35–39].

### B. Anabolic (revival of target tissue), Yang (tumorigenic arm): low energy consuming events-utilizing glycolysis (Warburg effect)

Following the release of oxidants toxins and utilization of high energy from mitochondrial OxPhos during destruction of foreign entity and the injured/infected target tissue, the activated cells simultaneously signal for polarization of immune cells (e.g., M2 or TAMs phenotypes, degranulated MCs) that would automatically shut-down mitochondria, allowing regeneration of TCA cycle intermediates (e.g., succinate–fumarate–oxaloacetate) and numerous other important mitochondrial activities [e.g., pyruvate-shuttle, pyruvate carrier proteins, synthesis of enzymes (e.g., SODs, cytochromes), metabolism of branched amino acids (leucine, valine, isoleucine) and synthesis of structural proteins and maintenance of mDNA synthesis] that are required for mitochondrial function. Yang (tumorigenic, post-inflammatory) events involve polarization of immune cells and non-immune pathways for expression of growth promoting mediators and decoy receptors [e.g., VEGF, IL-dRs, cortisol, epinephrine, superoxide dismutases (SODs), MMPs, PGE2, PI3 Kinases, mTOR, MAPK] to neutralize and remove the toxicities that are generated during Yin and to repair, reconstruct or remodel the target host tissue and terminate inflammation. Switching from oxidative phosphorylation in mitochondria, under hypoxic conditions and low energy utilization from glycolytic pathways are fundamental events that require differential bioenergetics (high-low energy switches from mitochondria to cytosol) for maintenance of effective immunity. The Yang processes are required not only for revival and repair mechanisms of injured tissue, but for mitochondrial recovery and biosynthesis of TCA cycle intermediates to generate efficient energy. Therefore, Yang events are naturally anabolic (low energy) and possess regenerative (growth) features of effective immunity, and function under low oxygen tension (hypoxia) for wound healing purposes.

Simply described, in an acute inflammation, apoptotic arm or Yin responses are catabolic processes requiring high energy consumption from mitochondrial OxPhos to express death signals, oxidants, enzymes and receptor molecules for destruction of both the enemy and the injured/infected host cells. In contrast, the wound healing or Yang responses are anabolic processes and require simultaneous polarization of immune and non-immune cells for generation of growth promoting factors to counteract and neutralize the toxicity of injured tissue. Yang responses follow mitochondrial shutdown and switch to cytosolic low energy utilization from glycolysis (Warburg effect) to resolve inflammation.

The major outcomes of an acute inflammation are lymphocyte-derived clonal expansion, increased synthesis of pathogen-, or allergen-specific antibodies and plasma and memory T and B cells that would prepare the body to defend and unleash appropriate responses such as antigen-specific antibody release when tissue is exposed to the same hazardous agents [5–7, 35–40].

The author’s original definitions of biphasic roles of Yin (tumoricidal) and Yang (tumorigenic) of effective immunity [35] present much larger applications for understanding the synchronized biorhythms and neuro-metabolic responses of sympathetic and parasympathetic systems that guard human health [5–7, 35–39].

## Bird’s eye view on differential energy requirements throughout life: violations of biological rhythms (laws) and induction of immune tolerance in tumorigenesis and angiogenesis

The crosstalk in Yin and Yang of effective immunity occurs continuously and simultaneously between and among cells, tissues, organs and glands (e.g., skin, liver, kidney, eyes, lung, heart, thymus, lymphoid organs, vasculature, neuronal, and gastrointestinal tract) for maintenance of health. In 2016, we proposed a working model for differential bioenergetics requirements of Yin and Yang pathways from fetus orderly growth, after birth and all the way to adulthood, aging process and development of chronic diseases and cancer [39]. The following sections briefly reflect further extension of the recently proposed model on analyses and integration of a large body of valuable scattered data pertaining to basic and clinical studies on developmental biology, immunity, aging, hormonal, metabolic and neuronal activities, to better appreciate induction of immune tolerance and cancer bioenergetics [5–7, 35–190]:

**Fig. 1:**
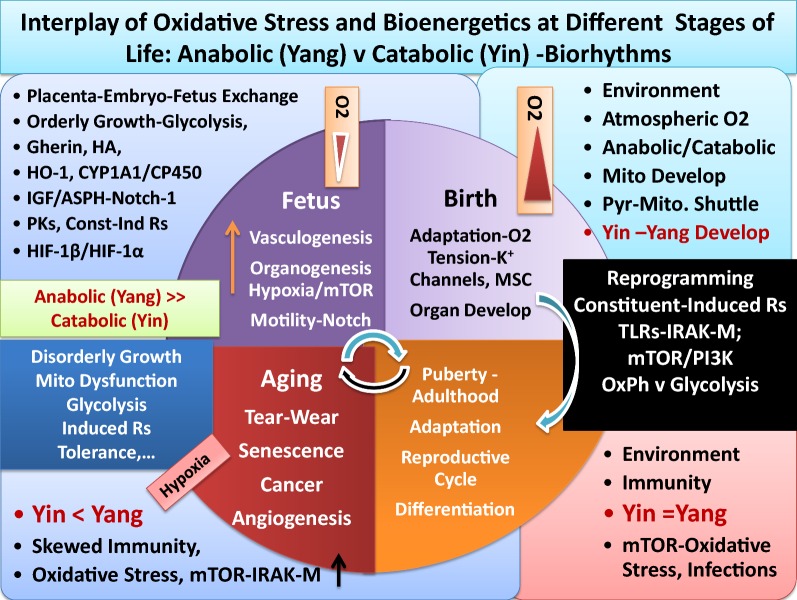
Schematic representation of Yin and Yang of immunity that parallels differential bioenergetics at various stages of life. It depicts that fetal orderly growth primarily utilizes glycolysis and constituent growth factors (e.g., IGF, mTOR) under low O_2_ and in the absence of mitochondrial development. After birth and exposure to atmospheric oxygen, major adaptation and reprogramming of organ systems including development of mitochondria and effective immunity (Yin–Yang) are required. Aging and oxidative stress is depicted to skew effective immunity including mitochondrial dysfunction, induction of tolerance and hypoxia that resemble anabolic conditions of fetal orderly growth, that could lead to disorderly growth of cancer cells (Yin <Yang). See text

**Fig. 2:**
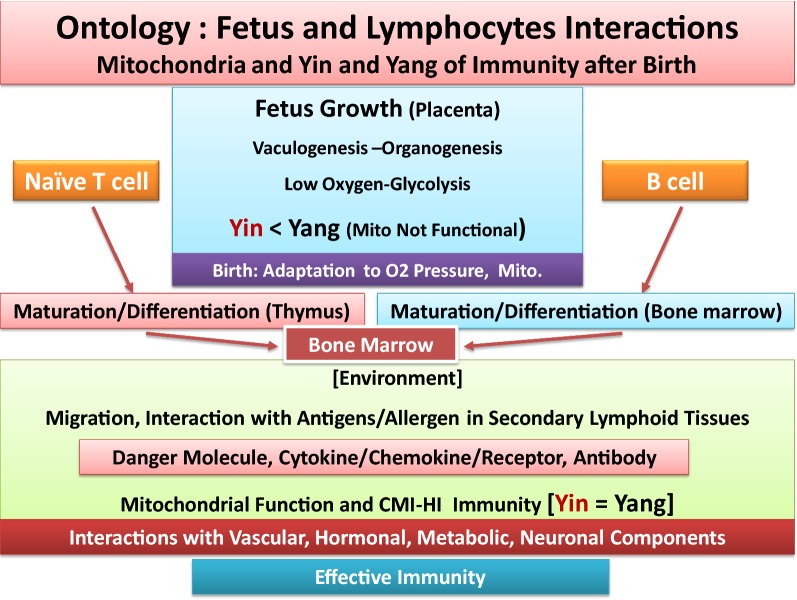
Schematic representation of ontology of fetal growth, showing vasculogenesis and organogenesis, under placenta’s limited oxygen tension. It depicts that fetus growth occur primarily in the absence of functional mitochondria and Yin (tumoricidal, high energy) arm of effective immunity. After birth and exposure to atmospheric oxygen and environmental hazards, adaptation, reprogramming and completion of organ development, formation of lymphoid organs, immune cell maturation and migration in thymus and bone marrow are required, for functionality of mitochondria and effective immunity (Yin–Yang). As depicted, requirements for differential bioenergetics and effective immunity, cell mediated or humoral immunity (CMI, HI), to defend body against harmful elements throughout life occur after birth. See text

**Fig. 3:**
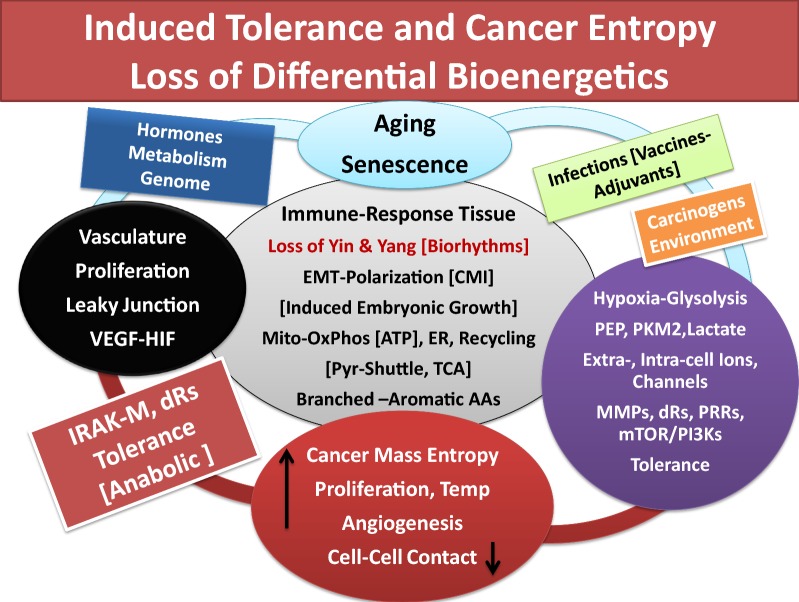
Schematic representation that aging (senescence) and oxidative stress lead to immune tolerance, associated with loss of differential bioenergetics (oxidative phosphorylation in mitochondria and Yin/tumoricidal arm of immunity), increased glycolysis, angiogenesis and hypoxia result in increased cancer cell growth and entropy. It depicts that in immune-responsive tissue loss of biorhythms (Yin–Yang) induces activation of embryonic (constituent) growth in epithelial cells and polarization of epithelium-mesenchymal transition. The events are accompanied by impaired pyruvate-shuttle and biosynthesis of structural proteins from branched or aromatic amino acids, increased IRAK-M and growth pathways (Yang, tumorigenic arm) such as mTOR/PI3Ks, VEGF, increased glycolysis to satisfy lawless growth of cancer cells, under hypoxic conditions and loss of cell–cell contact inhibition. See text

1. The body is a dynamic and energy consuming system involving highly complex interactions between and among multi-organs whose function continually evolves, from conception, placentation, embryonic and fetal growth, after birth, puberty and adulthood and aging or disease processes. The anabolic (regeneration, tumorigenesis or Yang) and catabolic (apoptosis, tumoricidal or Yin) arms of effective immunity follow highly organized biological circadian rhythms (biorhythms) requiring on–off switches of immune and non-immune responses that constitute the human health. Insufficient circadian rhythms (skewed biological clocks or loss of sympathetic or parasympathetic activities) are perhaps the result of one or more mutations or deficiencies in the circadian clock genes that influence the amazingly synchronized and interdependent features of biological oscillators in Yin–Yang or ‘effective acquisition time’ of immunity [5, 7, 39].
2. Growth requirements of embryo-fetus development are orderly processes (basically a one-way growth) occurring under protected environment of placenta during embryonic development involving expression of constituent receptors, projected from trophoblasts that anchor embryo and during fetus growth under limited oxygen tension (hypoxic condition). Angiogenesis and organogenesis, including development of lymphoid organs and generation of naïve pluriepotent stem cells or mesenchymal stem cells (MSC) require growth factors and metabolism, primarily using glycolysis for energy requirements. The energy production for growth involves constituent growth pathways such as expression of pyruvate kinases, aspartyl-asparginyl β-hydroxylases (ASPH or HAAH), mTOR/PI3Ks, insulin and insulin-like growth factor (IGF) [5, 39, 69–76]. The author proposed that the protection of fetus growth must occur in the absence of fully functional mitochondria and limited Yin (tumoricidal, growth-arrest) pathways to avoid necrosis that is dangerous to fetus survival and could lead to abortion [5, 39]. Thus, fetus orderly growth is practically established by anabolic pathways (Fig. 1). The growth-promoting events (Yang) are also required after birth for wound healing pathways and establishment of effective immunity. The overall features of fetus orderly growth include.
  - The earliest features in fetus growth are vasculogenesis and organogenesis, events that are completed and functional after birth [5, 39]. In the process of fetus growth, naïve or undifferentiated immune T and B cells are transported from placenta through fetus circulation to develop lymphoid organs. Being in the protective environment of placenta, fetus is not directly exposed to the atmospheric oxygen and is not challenged by the environmental hazards. The hypoxic conditions of placenta provide fetus natural/constituent tolerance. Therefore, there is no need for an effective immune surveillance (Yin and Yang), nor for functional mitochondria.
  - Majority of the required fetus constituent growth factors, receptors and enzymes (e.g., pyruvate Kinases, mTORs, hormones) also share features of wound healing, tolerance or tumorigenic (Yang) arm of effective immunity after birth.
  - Features of rapid aging and maturation in prenatal-embryonic tissues and postnatal development of childhood cancers [e.g., neuroblastoma, B-lineage infant acute lymphoblastic leukemia (ALL), mixed lineage leukemia (MLL), myeloid leukemia-Downe syndrome-ML-DS or medulloblastoma] have been reported [5, 39, 73–76]. The rapid childhood aging are associated with increased and progressive genomic mutations and instability and fusion, as evidenced by embryonic hyperplastic cell growth patterns that favor abnormal proliferations of cell survival under hypoxic conditions. Over expression of several genetically identified mutated growth factors (e.g., MYC, PI3K, MAPK, erythropoietin receptor-B cell factor-1-EBCR1 or BCR, Notch1, Notch2, FBXW7 and polymerases) or perhaps low level histamine [e.g., independent from MCs activation (‘leaky’ MCs), or in the absence of functional MCs] that trigger oncogenesis in childhood cancers are characteristics of adult cancers [5, 39, 73–76, 89].
3. After birth and during infancy the most pronounced biological changes and reprogramming relate to newborn’s exposure and adaptation to atmospheric O_2_ and completion of organs development. It is proposed that independent life of individual after birth requires the following major biological changes, adaptations and reprogramming (Figs. 1, 2).
  - Completion of mitochondria, the double membrane-bound organelles, for establishing the many biological events for production of energy and maintenance of bioenergetics. Among major pathways in mitochondrial development are the establishment of pyruvate-shuttle between cytosol-mitochondria, induction of pyruvate-carrier proteins, development of TCA cycle enzymes and generation of ROS that are byproducts of routine tissue metabolism (wear and tear), metabolism of essential branched amino acids (e.g., leu, isol, val) for biosynthesis of structural proteins and ribosomal recycling activities or perhaps chromatin remodeling-related events. It was proposed that biosynthesis of structural proteins after birth are required for maintaining architectural integrities and boundaries among and between cells and tissues (e.g., cell–cell-contact inhibition, vascular tight junctions, inhibition of epithelial–mesenchymal transition). Fully functional mitochondria have the capability for production of high energy (burst of ATPase hydrolysis) that are required by Yin events (e.g., activation of MCs, DCs, MΦs or T and B lymphocytes) at moment notice to combat foreign elements [5, 7, 39, 70–77, 82–119].
  - Exposure of newborn to outside environment and independent living demand establishment of fully functional biological circadian rhythms or the catabolic and anabolic responses in tissues. How the effective immunity and signal transduction between and among immune and non-immune pathways are completed after birth are among crucial knowledge gaps that deserve systematic, insightful and integrated understanding. It is likely that completion of many organs (e.g., lungs, heart, brain or gastrointestinal tract) functionality and reprogramming of tissues, cellular and subcellular components [e.g., mitochondria and TCA cycle, mucosal barriers (e.g., aryl hydrocarbon), transcriptional factors for numerous receptors and surface molecules, enzymes, epigenetic modifications, activation or protection of histone proteins and chromatin, recycling pathways in ribosomes, mucosal–flora interactions in gastrointestinal tract, maturation of immune and non-immune systems (biorhythms)] are not fully activated during orderly fetus growth. The molecular/cellular and subcellular adaptation and reprogramming could occur simultaneously after birth and at the interface between host and environment. The fact that during the first few months after birth, newborn limited immunity depends on mother’s immunity supports the above suggestions.
  - After birth, the complex establishment of effective immunity (fully functional Yin and Yang) and mitochondrial-related cellular functions are likely to take a few months to be completed. It is possible that the majority of components that contribute to catabolic pathways are genetically (innately) present as constituent elements but they are not functional or not expressed until after birth. Potential examples of such constituent pathways are structurally naïve immune and none immune cells, sympathetic and parasympathetic neuronal systems, inactive/inhibited apoptotic factors and components of double membrane in mitochondria. Examples of constituent components that are likely to be functional after birth include immature or naïve lymphocytes, oxidases, pro-inflammatory cytokines, toxins, pyruvate-shuttle, pyruvate carrier proteins, TCA cycle proteins/enzymes, metabolic pathways of essential amino acids (e.g., leu, ileu, val) and biosynthesis of structural proteins for cell–cell contact inhibition and vasculature tight junctions, components of epigenetic modifications, hypo- or hypermethylated genomic materials.
4. It is noteworthy that biological development and reprogramming and responding to environmental conditions continue through puberty for hormonal regulations and reproductive cycles and adulthood; many of which processes decline or change during aging process (senescence).
5. Continued proton pumping and generation of electricity across the membrane, are crucial for establishing pH gradients and differential acidity among extra-, and intra-cellular membrane components and cytoplasm for numerous routine biological activities [e.g., transport of solutes/osmolytes and nutrients, stimuli-induced expression of danger signals and activation of immune cells, degradation and growth-arrest of defective cells (e.g., cancerous cells), inappropriate synthesis of proteins and mutated DNAs/RNAs, detection and destruction of pathogen’s structural components, immune cell recognition and activation, proliferation, wound healing and growth, lysosomal activities for digestion and recycling of proteins and lipids] as well as, numerous biosynthetic pathways in mitochondria. It is likely that the extent of proton pumping provided through vascular or cellular membrane by ATPases or exchangers (e.g., Na^+^/H^+^ exchanger, Ca^2+^/ATPase) alter, to varying degrees, from the time of fetus growth, after birth, during reproductive period and adulthood, in aging or disease processes (e.g., neurodegenerative and autoimmune complications or carcinogenesis).
6. Intrinsic or extrinsic components that are recognized as foreign agents (e.g., pathogens, allergen, cancerous and defective cells, useless proteins, lipids, low level carcinogens or pathogen-specific vaccines and ingredients/adjuvants), as well as, aging process, could temporarily or permanently disturb the effective membrane potential or pH gradient (proton pumping) across the extracellular or intracellular membranes that would skew signal transductions and auto-regulatory processes of cellular biorhythms [5, 7, 15, 20, 36–39, 45, 82, 83, 89, 140, 151, 179–185].
7. Innocuous substances and occasional exposures to a wide range of foreign elements are ordinarily and differentially ignored by tissues that are immune-responsive or immune-privileged (natural immune tolerance). The immune-privileged tissues (e.g., CNS, BBB, avascular cornea, neuroretina, reproductive organs) are highly sensitive toward oxidative damage. These tissues possess special anatomical or molecular features to minimize response to oxidative damage [5, 7, 36–40, 89]. These tissues present higher levels of tolerance compared with the immune-response tissues (e.g., epithelium, endothelium, mucus-secreting goblet cells). However, the levels of immune tolerance are limited in both types of tissues [5, 36, 37]. Persistent tissue stimulation and exposure to potent pathogens or treatment of patients with combination of radiation and chemotherapy that are claimed as ‘targeted’ therapy, ‘personalized’ or precision’ medicine, using potent apoptotic factors, monoclonal antibodies against specific growth factors, are deemed hazardous (biological terrorists) to the immunity. These conditions stimulate a wide range of immune responses causing ‘mild’, ‘moderate’ or ‘severe’ acute or delayed hypersensitivity reactions (immune disorders) that could lead to manifestation of different diseases, organ dysfunction, multiple organ failures (MOFs) or death [5, 7, 35–39, 43, 89].
8. Oxidative stress and alterations of Yin and Yang properties of effective immunity often differentially facilitate growth promotion (anabolism) or growth arrest (catabolism) in different tissues. The processes are natural/inherent properties of immunity (biorhythms) to resolve inflammation. Potential adverse influence of extensive oxidative stress in immune-responsive tissues includes epithelial–mesenchymal transition (EMT), changes in extra- and intra-cellular membrane matrix [e.g., MMPs, IV collagen biosynthesis, cellular transport activities, aqueous charges (altered H bonds)] further affecting cellular hydrophobicity or hydrophilicity, protein foldings and cellular function. Extensive oxidative stress is the results of exaggerated expression and co-expression of growth and apoptotic factors that result in immunological chaos (immune tsunami) that could damage the tissue integrity at multiple levels of biological, mechanical, physical and bioenergetics. Serious damages in immune-responsive tissues lead to changes in bioenergetics and metabolism and proton pumping in the direction of initiation of tissue growth, neoplasia, precancer-polyps, invasive cancer growth and angiogenesis. In immune-privileged tissues, exaggerated expression of tumoricidal mediators (catabolic or apoptotic factors) causes necrosis and local immune responsiveness in tissues in the direction of neurodegenerative and autoimmune diseases (e.g., Alzheimers, Parkinson’s, multiple sclerosis, atherosclerosis).
9. Glucose toxicity-induced changes in immune response dynamics could additionally and adversely influence tissues that are insulin-dependent (e.g., muscle, liver, adipocytes) or insulin-independent (e.g., vasculature, BBB, retina, cornea, kidneys) for glucose transport, metabolism or growth as contributing factors in the induction of tolerance and initiation of chronic diseases such as diabetes and cardiovascular complications, hypertension, stroke, as well as increased risk of carcinogenesis [5].
10. Aging processes (immunosenescence) induce minor or major alterations in immune function [e.g., changes in age-induced cell death (AICD) and damage-induced cell death (DICD)] and enhance the vulnerability of host tissue toward loss of balance between apoptosis and wound healing processes, increased memory B or T cells, clonal expansion and increased hypersensitivity of humoral or cellular responses toward new or old stimuli (e.g., histamine intolerance, autoimmunity), additionally contributing to the mitochondrial and ribosomal dysfunction (mitophagy, autophagy) [5, 7, 35–39, 52, 53, 59–61, 65–68, 83, 88, 89, 109]. Longevity and continuous exposures to microbiota or pathogen-specific vaccines and adjuvants/ingredients (e.g., mercury, aluminum phosphate, aluminum hydroxide, l-histidine, embryo serum) to varying degrees, could induce polarization of immune cells (e.g., MΦs, MCs, DCs, T or B cells) and direct expression of wound healing factors, decoy or pattern recognition receptors [e.g., IL-1dRs, IRAK-M), surface molecules (e.g., CD-11, CD-73), low level circulating histamine (independent from antigen-specific MCs IgE-fcεR aggregation and degranulation)] ([5, 7, 35–39], manuscript in preparation).
11. As noted above, the growth promoting events often occur under hypoxic conditions and low energy consumption from glycolysis. The conditions are characteristics of Yang pathways during normal fetus growth or wound healing events and neovascularization as well as, cancer growth and angiogenesis. Therefore, induction of tolerance, while a feature of effective immunity (Yang, anabolic or growth-promoting), under oxidative stress represents extended wound healing processes. Continued activities of Yang (tumorigenic) are associated with mitochondrial dysfunction (mitophagy) causing damages to tissue recycling processes and ribosomal activities (autophagy) that are features of skewed immune responses or immune suppression (Figs. 1, 2, 3).
12. In immune-responsive tissues, enzymes or other factors that modulate senescence negatively regulate differentiation of tumor cells (mitosis) through epithelial–mesenchymal-transition (EMT) often involving polarization, unscheduled or immature synthesis and activation of immune cells, or expression of factors and receptors [e.g., TGFβ-R1, peroxidasin (PXDN), collagen IV], selective membrane catalysis of sulfimine or changes in H bonds with protein structures that create “dual negative feedback loop” and further influence tissue metabolism, fibrosis or cancer.
13. During EMT, polarized immune cells are capable of expression of a panel of growth-promoting factors and decoy receptor molecules that are immune suppressive and signal for mitochondrial shutdown, inhibiting pro-inflammatory responses, leading to tolerance in favor of tumorigenesis and enhanced activities of cytosolic glycolysis.
14. Stimuli-induced activation and polarization of immune cells are accompanied by activation of several other metabolic pathways and expression of factors [e.g., carbonic anhydrase 2 (CA2), constituent or induced pyruvate kinases (PKM1, M2), phsosphoenol pyruvate (PEP), PEP carboxy kinase (PEPCK)] with potential different bioenergetics requirements that could influence cellular function. For example, changes in PKM1 and PEP in red blood cells, whose principal energy source is from glycolysis could lead to altered hemoglobin metabolism, anemia, hemophilia, hemolysis, cardiovascular complications, jaundice (bilirubin) and blood-related diseases including cancers (e.g., hepatocellular carcinoma) or severity of Guillian–Barre syndrome ([5], manuscript in preparation);

In summary, disturbance of the well orchestrated crosstalk in effective immunity by frequent exposures to immune disruptors and aging processes cause minor or major degrees of retardation in immune response dynamics that could increase the risks of initiation and progression of a wide range of ‘mild’, ‘moderate’ or ‘severe’ immune disorders in susceptible tissues. Low level circulating histamine was proposed as a blueprint in the induction of tolerance leading to diverse immune disorders and alterations of acid–base balance and tissue bioenergetics [5, 39]. Cancer was hypothesized as a ‘severe’ accumulation of delayed hypersensitivity responses in tissues. Oxidative stress-induced skewed biological circadian rhythms and dysfunction of mitochondria (mitophagy), peculiarly provide opportunities for cancerous cells to utilize enhanced activities of cytosolic glycolysis pathways for consumption of energy from Crabtree or Pasteur Effects (induction of 'dark energy'), conditions that are toxic to normal cell survival, but facilitate cancer cells lawless proliferation and increased entropy (see below).

## Differential mitochondrial bioenergetics requirements in Yin–Yang of immunity: oxidative stress-induced chronic diseases and cancer

The principal free energy source that is required for all body’s biochemical pathways is the universally known molecule of ATP. In general, the normal function of organ system uses ATP as both an intracellular energy source and an extracellular messenger for energy requiring transmission of signals in CNS or optic nerve or other immune and non-immune cells for exocytosis, formation of Golgi complex, delivery of vesicles, as well as active transport of solutes/osmolytes and nutrients (e.g., glucose, Ca^2+^, ascorbate, myo-inositol), enzymatic processes (e.g., oxidases, kinases, lipases, hydrolases), protein biosynthesis and other physiological pathways, and physical, mechanical or locomotion and architectural integrity of cells/tissues/organs (Figs. 2, 3) [5, 7, 35–39, 78, 82, 83, 89, 101, 139, 140, 142–150, 178–220].

Mitochondrial dysfunction (mitophagy) has been linked to a number of age-associated and chronic health problems, including migraine, cardiovascular and neurodegenerative diseases, sarcopenia, infertility, kidney and liver diseases, cancer, drug toxicities and other illnesses that often accompany fatigue syndrome [5, 7, 35–39, 91, 142–150, 178–190]. Observations on the association between defects in energy metabolism of cancer cells and effective respiration (oxidative phosphorylation) and the abnormal rates of aerobic glycolysis for ATP synthesis that was originally reported by Otto Warburg, led to mitochondrial injury/damage and concept of mitophagy [5, 40, 53–55, 91, 184–190]. It is now well documented that cancer cells are capable of converting glucose into lactate and pyruvate. Synthesis and diffusion of lactate create acidic conditions in the extracellular matrix (e.g., MMPs) and change the integrity of membranes (e.g., IV collagen biosynthesis or receptor molecules) as contributing factors that alter tissue metabolism and bioenergetics during growth of cancer mass, invasion and metastasis. These and other important observations on the metabolism of cancer cells and the role that mitochondrial dysfunction play in cancer growth evolved in two hypotheses of survivability and adaption of cancer cells as ‘Crabtree Effect’ and ‘Pasteur Effect’, the glucose triggering mechanisms for cancer proliferation as outlined below [5, 39, 185–190].

- *Crabtree Effect* Tumor cells and normal proliferating cells or pathogens (e.g., bacteria or yeast) have limited respiration in the presence of high glucose concentration. The phenomenon is known as Crabtree Effect. Under such conditions, cancer cells are able to trigger the competitive inhibition of oxidative phosphorylation (respiration) for using phosphate groups (Pi, inorganic phosphate) and ADP, through glycolysis for their enhanced growth requirements, conditions that are toxic to normal cells. An excellent publication by Hammad et al. [189] explains the Crabtree and Warburg Effects and the roles that glucose and rate-limiting steps in constituent kinases (e.g., pyruvate kinases, phosphofructokinase) play in regulation and uptake of substrates within and outside mitochondria for control of ATP production and mitochondrial intermediates. While detailed mechanisms of the effects are debatable, it seems that the abundant presence of glucose, perhaps including hyperglycemia of diabetes, impair mitochondrial normal function at several levels (e.g., inhibition of energy requiring steps in pyruvate-shuttle and subsequent events in carrier proteins and enzymes that are needed for biosynthesis of TCA cycle intermediates) and energy production. The availability and activation of other factors (e.g., adenosine, histamine) or changes in acid–base homeostasis during production of lactate and dissipation of energy could be interdependent contributing factors in slow-down of mitochondrial function. While enhanced glycolysis and glucose uptake by cancer cells favor promotion of lawless growth of cancer masses, the conditions could create differential entropy that adversely affect the surrounding tissues (see below) [5, 7, 38, 39, 184–189]. It is suggested that Crabtree Effects initially share some features of Yang arm of immunity during normal wound healing or during orderly growth of fetus that occur under hypoxic conditions; when mitochondria are not fully functional. These and related metabolic pathways, if studied systematically, should provide unique opportunities and challenges to efficiently target and control the growth of cancer cells.
- *Pasteur Effect* As originally described by Otto Wargurg, the tumor cells are able of inducing glucose utilization and conversion to lactate in the presence of oxygen, a phenomenon called Pasteur Effect that diminishes glycolytic metabolism in yeast [5, 39, 55, 92, 189]. The utilization of glucose oxidation and conversion to lactate perhaps play important roles in metastasis (diffusion to extracellular matrix and acidity) and enhanced proliferation. The above observations and concepts argue that cancer cells have higher rates of consumption for either or both oxygen or glucose. That means when concentration of either nutrient is reduced in the microenvironment of cancerous tissue, cancer cells can thrive, while normal cells cannot. Factors that are toxic to normal cell survival but facilitate metabolic adaptability of cancer cells to microenvironment include the followings [5, 47, 48, 53–55, 90, 189]:
- Increased mutations or damage in mitochondrial DNA and altered pyruvate-shuttle or pyruvate carrier proteins.
- Elevation of hexokinase (1,6 phosphofructo kinase) activities.
- Lysis or loss of mitochondrial cristae structures and altered mitochondrial protein and lipid content.
- Increased acidity (lower pH) in extra-, or intracellular environments (presence of lactate) and altered hydrophilic or hydrophobic properties of channels for transport of cationic or anionic molecules and proteins and aquaporins.
- Potential utilization of mitochondrial cardiolipin as additional sources of energy.
- Loss of cell–cell contact inhibition.
- Induction of local entropy and higher temperature in cancer masses compared with surrounding tissue.

Detailed analyses of related data suggest that cancer oncogenes mutations and growth dysregulation, along with associated enhanced expression of PI3K/Akt and altered balance in c-MYC, HIF or p53 pathways, adversely influence transport and metabolism of glucose, amino acids, ions or water channels (aquaporins) of the surrounding normal tissues that impair extra- and intracellular components (e.g., mitochondria, ER, nucleus). For example, loss or leak of cardiolipin, a key mitochondrial lipid, located in the inner membrane of mitochondria has been demonstrated in damaged mitochondria in cancer environment [5–8, 39, 53–55, 60, 81–84, 102, 143, 144, 152, 166, 174, 178–191]. Cardiolipin is among several other necessary components that are needed for efficient cellular respiration and maintenance of chemiosmosis (aquaporin channel). While details of the role of mitochondria and inflammation in diseases of aging are not well understood, it is likely that acute inflammation initially causes a burst of energy (ATP hydrolysis) in mitochondria of activated immune cells to generate sufficient energy for induction of oxidants and toxins during apoptosis (Yin). In the process, cardiolipin molecules oscillate/move from the inner to outer mitochondrial membrane and signal to facilitate termination of inflammation during wound healing (Yang) events and to preserve regeneration and function of mitochondrial TCA cycle intermediates.

## Exocytosis, Ca^2+^ fluxes and recycling activities in lysosomes: anabolic and catabolic energy requiring events

Induction and completion of acute of acute inflammatory responses always require participation of lysosomes and proper recycling of proteins and lipids of phagocytised materials, expression of lysosomal hydrolases and proteases during autophagy. Among numerous biological alterations that occur under oxidative stress and aging, the body’s ‘self-eating’ processes of lysosomes and associated impaired activities in mitochondria or Golgi play crucial roles in homeostasis of immunity or immunosenescence, chronic diseases or cancer [5, 39, 88, 89, 126, 184, 220]. For example, the early events in stimuli-induced activation of MCs via receptor (FcεR) aggregation and degranulation (pharmacological effects) require membrane fusion for exocytosis of granules. The processes involve secretory lysosomal activities and expression of several protein receptor molecules, at different stages of membrane fusion and require ATP hydrolysis and mobilization of Ca^2+^ from intracellular stores. Lysosomal exocytic activities are triggered following an increase in free Ca^2+^ (cation) concentration, acidified by H+/ATPase hydrolysis for fusion and flux at the plasma membrane level [5, 39, 88, 89, 184, 220]. The complex pathways during degradation, clearance and recycling of proteins require energy-dependent biosynthesis of a number of acidic hydrolases or proteases and surface molecule, signaling within lysosomes, endoplasmic reticulum (ER), Golgi apparatus and mitochondria [5, 38, 39, 88, 89, 92, 143, 144, 150, 152, 169, 171, 180–184, 192]. These interdependent catabolic and anabolic activities are universal in living cells and influenced by aging and disease processes. Data using inhibitors of MCs degranulation, antihistamine agents, oxidative phosphorylation or glycolytic pathways (e.g., glucose or pyruvate oxidation), under a wide range of immune dysfunction or carcinogenesis suggest that MCs activation and release of histamine and other preformed or newly synthesized mediators, require activation of lysosomal exocytosis and Ca^2+^ flux from the stored-operated Ca^2+^ channels as part of effector function of MCs. Use of antihistamine or anti-allergic agents (e.g., oxatomide, astemizole, olopatadine) in the in vitro models of allergies or basophilic leukemia cells (RBL-2H3) seem to suppress one or a combination of interdependent MCs responses such as inhibition of AA pathways, cytokines (e.g., IL-4) or Ca^2+^ influx through receptor-operated channels (ROC), phosphorylation of P38 mitogen-activated protein kinase (MAPK) and c-Jun NH2-terminal kinase, pathways that are involved in IL-4 gene expression and tumorigenesis (anabolic pathways?).

Inflammatory conditions such as potent pathogen-induced severe immune reactions (e.g., sepsis, meningitis, pneumonia, anaphylaxis), major trauma as well as, the claimed cancer ‘targeted’ drugs, ‘personalized’ or ‘precision’ medicine (in combination with partial or total body radiation) that are potent apoptotic factors or monoclonal antibodies induce a vicious cycle of immune cell activation (immune tsunami or cytokine storm) [5–8, 39, 43]. Potent immune disruptors require high energy demands not only from local tissue mitochondria, but they also cause induction of systemic or intraperitoneal exaggerated expression of pro-inflammatory cytokines and toxins such as ROS, caspases, oxidases, damage-associated molecular pattern (DAMP), increased C-reactive protein (CRP), altered muscle F-actin filaments and high-mobility group box 1 (HMGB1). Drug-induced accumulation of inflammatory responses often cause serious damages to the function and integrity of tissues and multiple organ failures (MOFs) in muscle, liver, kidney, lung, brain and heart and patient’s death. Therefore, stimuli-induced frequent expression and co-expression of growth-arresting (Yin, tumoricidal) and growth promoting (Yang, tumorigenic) cytokines such as TNF-α, ROS, interleukins (e.g., IL-4, IL-6, IL-8, IL-10, IL-12,IL-18), monocyte chemoattractant protein 1 (MCP-1), CXCR3, neutrophil extracellular traps (NETs), abnormal expression of vasculature components (e.g., P- and E-selectins, ICAM-1, VCAM-1) damage mitochondrial oxidative metabolism and DNA [5–8, 35–43, 169–175, 178–194].

## Definitions of constituent (Innate) and induced (‘Designer’) pattern recognition receptors (PRRs) in health and diseases

Pattern recognition receptors (PRRs) and surface molecules play crucial roles in signal transduction mechanisms and contribute to all aspects of cells/tissues functions such as visual transduction, bone and lipid biosynthesis, bioenergetics, cellular trafficking and infiltration, differentiation and growth, nuclear/chromosomal or chromatin activities, neuronal pathways, tissue necrosis or growth in immune and non-immune systems for maintenance of health or induction of diseases [5–8, 35–39, 191–202]. Discussion on the time course kinetics and mechanisms of actions of receptor molecules presents a complex molecular universe with unique or shared features that is beyond the scope of this perspective. Suffice to note that defining only the roles that insulin receptors play in health or induction of major diseases such as diabetes and cardiovascular complications or carcinogenesis is a huge topic and yet to be fully understood [5–8, 39, 89, 202–207]. Analyses of a wide range of receptors or pattern recognition molecules (‘biological signatures’) that were defined as constituent or designer receptor molecules [5] are outlined below to better appreciate the crucial roles that these molecules play in maintenance of health or induction of tolerance in carcinogenesis: [5, 39, 126, 174, 202–208, 212–215].

### Constituent: innate and adoptive receptors and surface molecules

Constituent receptors and surface molecules are essential (innate) members of the embryonic growth and development. They are also synthesized and/or regenerated after birth for routine maintenance of enormous biological activities and molecular schedules of organs/tissues functions throughout life. Constituent receptor molecules are involved in extracellular and intracellular signaling, actions of hormones, metabolites, cytoplasm or nuclear and genomic transporters/enzymes, mitochondrial membrane trafficking, carrier proteins, neuronal transporters, or related surface molecules that routinely contribute to the physiology of immune or non-immune systems. Examples of such constituent pattern recognition receptors are the various insulin receptors for glucose transport or metabolism in insulin-dependent (e.g., muscle, adipocytes or liver) or insulin-independent tissues (e.g., vasculature, brain, neuronal tissues, retina, kidneys). These receptors are required during fetus growth and organ development and for maintenance of health throughout life [5, 7, 39, 125, 126, 130, 151, 157, 158, 171, 202–207].

After birth, each of the genetically-determined receptor molecules are strongly influenced by the signals they receive from the environment and become adaptable or programmable to the quality of nutrition (initiated from mother’s milk or consumed baby formula) and exposures to a variety of bioactive agents, microorganisms, environmental chemical and biological hazards. Modifications of constituent receptor molecules that occur after birth seem to parallel the development of mitochondria and Yin–Yang of effective immunity as infant becomes independent from the protective environment of placenta and requires adaptation and reprogramming to the atmospheric oxygen (see above). Major changes in constituent receptors occur in the gastrointestinal and upper respiratory tracks, skin or perhaps ocular tissues. Therefore, constituent/innate receptor molecules are capable of maturation or adaptation to gene-environment interactions and interdependent shifts or synergies with induced receptor molecules for maintenance of health or induction of diseases.

### Induced, ‘Designer’ or pattern recognition receptors (PRRs) and surface molecules

‘Designer’ or induced receptors often present transient functions when tissue is exposed to specific stimuli (e.g., allergen, pathogens, certain foods, carcinogens, biological, chemical or environmental hazards) [5, 39]. Stimuli-induced specific toll-like receptor molecules (TLRs-1-9) that signal for expression of specific cytokine and chemokine receptors or antibody bindings (e.g., IgE-fcεR, IgGγRs, IgARs) and related surface molecules are examples of ‘designer’ or induced receptor or surface molecules during sensitization or activation of immune cells. Receptors with short half-lives may fit the profile of either or both constituents and induced. These receptors include a wide range of molecules for ion channels, including activation-induced enhanced Ca^2+^ permeability, expression of receptor potential channels such as transient receptor potential cation channel, subfamily 4 (TRPM4), TRPC 5, in combination with STIM1 and CRACM1 that contribute to FcεRI-induced Ca^2+^ influx during MCs degranulation [5, 39, 99, 101, 151, 157, 158, 171, 182].

### Pattern recognition receptors (PRRs) and induction of immune tolerance in multistep carcinogenesis

Initiation of immune responses in antigen presenting cells (APCs) toward microbials/pathogens or defective cancerous cells (foreign elements) is mediated through a number of pathogen recognition molecules or receptors including specialized toll-like receptors-TLRs (e.g., TLR1-9). Stimuli-induced expression of TLRs contributes to the differential recognition of molecular structures or sub-structures of pathogens for specific sensitization and activation of APCs and appropriate response. The important roles that TLRs, decoy (dRs) or pattern recognition receptor molecules (PRRs) play in defense of body for activation of innate or adaptive immune or non-immune pathways for generation of death factors, pro-, and anti-inflammatory responses for human development or initiation or termination of acute or chronic inflammatory processes, immune tolerance and cancer have been extensively studied [5, 7, 39, 67, 72, 79, 157–162, 191, 194–207, 210–217].

Decoy receptor molecules are agonist-binding proteins that sequester inflammatory cytokines and signaling receptor components during termination of acute inflammation (Yang). Structurally, decoy receptors are incapable of participating in signaling receptor complexes. Decoy receptors act as promoters or inhibitors of proliferation of immune and non-immune cells and contribute to host immune homeostasis or induction of ‘tolerance’ or ‘intolerance’ in ‘mild’, ‘moderate’ or ‘severe’ immune disorders or multistep carcinogenesis [5, 39]. The cytokine receptor dual function (decoy behavior) was originally defined for IL-1 (IL-1dR) and IL-2 (IL-2dR) receptors. Briefly, decoy or specialized receptors and related surface molecules are involved in a wide range of biological activities such as binding to immunoglobulins/antibodies (e.g., MCs-IgE-FcεR; MΦRs, IgM Rs or IgGRs, mucus-secreting IgARs), histamine (HRs) or surface molecules (e.g., CD 11, CD22, CD40,CD80, CD83, CD86), CD86, CAIX, integrin, TNF-Rs, IL-1Rs, indolamine 2,3 dioxygenase, CAMs, ECM). Other receptor molecules that are able to sequester ligands and participate in termination of inflammation or proliferation of immune or non-immune cells or cancer growth and angiogenesis include receptor molecules for MCSF, iNO, PGE2 and/or low level histamine [5, 7, 39]. Some decoy receptors such as IL-1dRs have been identified for regulation of other cytokines such as IL-8, a member of the IL-1 family, and TNFR superfamily (e.g., osteoprotegerin) [5, 39, 79, 191–196, 209–217].

An extensively studied cytokine and decoy receptor function that is involved in inflammation and multistep carcinogenesis is tumor necrosis factor-alpha (TNF-α), a cysteine-rich cytokine and its receptor molecules (TNFR-1, TNF-Rp55, TNF-Rp75). The TNFRs act as transponders of TNF by receiving and transmitting signals and are able to trigger several biologically different functions during Yin and Yang of acute inflammation (circadian biorhythms) for maintenance of tissue homeostasis and elimination of host cells with damaged DNA. A wide range of immune disruptors, extrinsic/exogenous or intrinsic/endogenous are able to induce synthesis and production of TNF-α and its receptor molecules in a variety of cell types [e.g., MΦs, T cells (Th1, Th2), DCs, MCs or keratinocytes]. TNFRs also have proliferative capabilities for growth of fibroblasts or thymocytes and induction of expression of mitochondrial or cytoplasmic superoxide dismutases (SODs) to terminate acute inflammation (Yang) during wound healing [5, 39, 126, 191, 194, 196, 209–211].

Toll-like receptors (e.g., TLR1 and TLR2) are known to recognize and bind to ligands of specific molecular patterns of microbioms such as the tri-acyl lipopeptides, lipoarabinomannan or bacterial wall peptidoglycan (PGN) and lipoteichoic acid (LTA), and phospholipomannan of the bacteria (e.g., mycobacterium tuberculosis, *Staphylococcus aureus*). For example, LPS-induced expression of TLRs leads to activation of immature DCs and differentiation and migration of appropriate phenotypes from peripheral tissues to lymphoid organs to activate naïve T cells (T0) and upregulate expression of major histocompatibility complexes (MHC I and II) and co-stimulatory surface molecules. The actions are followed by expression of other pro-inflammatory cytokines and chemokines for destruction of microbial (Yin, catabolism). The actions require high energy production from OxPhos in mitochondria. Stimuli-induced expression of TLRs is associated with induction of IL-1R that form a group of superfamily regulatory proteins with shared and special features for signaling to intracellular domains to induce expression of other cytokines at extra-, and intra-cellular levels and for recruiting adaptor molecule response such as myeloid differentiation (MY 88 gene) and formation of receptor-adaptor complex domains for proper immune responses [5, 35–39, 83, 201, 212–215].

Review of data on bacterial or viral infections on models of liver injury or influenza infection-induced lung tissue damage and lysis of epithelial cells shows exaggerated immune cell activation and increased expression of interleukin (IL) receptor-associated kinase M (IRAK-M) to protect tissue damage [5, 35–39, 190–201]. These and related data suggest the limited immunopathology protection of IRAK-M in tissue without influencing or decreasing the viral clearance. Therefore, it seems that expression of IRAK-M protects, to some degrees, damaging the lung, or perhaps other epithelial tissues, and preventing complications of asthma. Expression of IRAK-M also limits neutrophil-induced damage to tissue while improve tissue remodeling. Data on TLR7-induced MΦs activation (Yin, M1 phenotype?) and tolerance (Yang, M2, TAM phenotype?) seem to accompany an elevated expression of IRAK-M, decreased expression of TNF-α, followed by expression of NF-κB, p38 and stress-activated protein kinase (SAPK) within the protein family of mitogen-activated protein kinases (MAPKs).

Induction of tolerance involves increased expression of IRAK-M and sulfhydryl domain-(SH2) containing protein-tyrosine phosphatase (SHP-1) activities and 2 subgroup of MAPKs, c-Jun-NH(2) N-terminal kinase (JNK) and p38 MAPK pathways that signal for immune suppression. Depending on the extent of oxidative stress these events that signal for wound healing are potentially involved in temporary or permanent dysfunction of mitochondria to avoid oxidative damage to tissues [5, 39, 190–200]. Involvement of SH-containing proteins in IRAK-M activity, acting as anti-oxidants and scavengers of free radicals (oxidants), supports mechanisms of termination of inflammation (Yang events) that we described for acute inflammation [5, 36–40]. Lagler et al. [198] demonstrated that activation of TREM-1 during the early *Streptococcus pneumonia* infection resulted in a decreased expression of lung IRAK-M and elevated pro-inflammatory cytokines, suggesting that low expression of IRAK-M is mediated by TREM-1 to promote efficient early bacterial clearance. Therefore, ‘tolerization’ and the associated expression of IRAK-M may act to prevent liver cell death. LPS-induced expression of TNF-α was associated with a lack of IRAK-M induction in liver cirrhotic lymphocytes [196].

Similar reports demonstrate that the mannose receptors and C-type lectin-induced DCs’ specific intercellular adhesion molecule-3-grabbing non-integrin (DC-SIGN) binds to *Mycobacterium* tuberculosis cell wall components called mannose-capped lipoarabinomannans (Man-LAMs) [213]. Furthermore, Man-LAMs inhibit LPS-induced IL-12 p40 production, NF-κB activation and IRAK-1-TRAF6 interactions.

The action is accompanied by increased IRAK-M expression [5, 79, 213].

The author suggested that the molecular complexes associated with immune suppression for induction of tolerance via IRAK-M, if not the same as decoy receptors (e.g., ILdR) that are expressed during polarization of immune cells (Yang pathways), they operate on similar or complementary regulatory processes and follow similar biological principals for maintenance of health or initiation of diseases [5].

## Cancer; an induced disease of twentieth century facilitated by decision makers! Role of virus-contaminated polio vaccines and sharp increased in cancer incidence and mortality and other diseases. Creation of a sick drug-dependent society for corporate profit

In this section, it is important to first remember that the role of pathogens (viruses, parasites and bacteria) in the induction of acute or chronic inflammatory and infectious diseases or cancer has been documented for over a century [2, 5, 7, 10, 20–24, 39, 40, 56, 122, 123, 128, 155, 169, 216]. Secondly, while in the last century advances in development of antibiotics, better hygiene and modern technologies improved longevity, the aging populations in America (current baby boomers) and the younger generations are not healthier compared with the previous generations at the same age [5, 7, 16, 20, 39, 175, 176, 219, 222–224]. In 1900s, the estimated risk of cancer was one in every 20 individuals (5%). The rate coincided with the normal low risk of cancer as a hereditary disease in the general population. Furthermore, in 1940s (four decades later), before vaccinating the American public with virus-contaminated polio vaccines, 1/16 individuals (approximately 6%) developed cancer (1% increase in cancer incidence over 4 decades), according to available statistics. However, since 1955s/1960s after public (current baby boomers) consumed virus-contaminated polio vaccines (injection or ‘sugar pills’), the cancer incidence and mortality and numerous other neurological and autoimmune diseases sharply increased [5, 7, 16, 39, 79, 89, 175, 176, 219, 222–224]. In 2013 (six decades after initial consumption of contaminate vaccines), the American Association for Cancer Research (AACR) announced that 1/3 (33%) of all women and 1/2 (50%) of all men develop cancer in their life time; that is up to 10 folds increase in the deadly incidence of cancer in the last six decades! [5, 7, 16, 20]. In 1955, decision makers in medical/cancer established ignored the existing data that viruses cause cancer. They also downplayed the serious safety concerns and warnings of a competent and devoted professional at NIH (Bernice Eddy, MD, microbiologist) who discovered that Sabin polio vaccines (prepared in monkeys’ kidneys) were contaminated with live viruses [e.g., simian virus (SV-40) and other filterable viruses]. This American tragedy significantly damaged the health of the last 3 generations in America and to lesser extent, health of other developed nations who consumed the contaminated vaccines [5, 7, 11, 13, 14, 16]. Since the 1955s, the immunity of old and young have been further weakened by heavy publicity to inoculate the individuals with other pathogen-specific vaccines and their unhealthy ingredients and adjuvants (e.g., Swine flu, HPV, hepatitis B or C, measles, meningitis, EBOLA, herpes) or even BCG, whether or not the vaccines are contaminated with live pathogens. The younger generations who are also exposed to a wide range of biological and environmental hazards or low level carcinogens suffer from a wide range of immunological disorders (e.g., allergies, asthma, neurological and autoimmune diseases), conditions that are features of age-associated chronic illnesses [5, 7, 11, 12, 15–25, 74, 78, 89, 155, 202, 223–225, 246].^6^, ^7^, ^8^, ^9^ While the decision makers insist that cigarette smoking is the major factor in the increased risk of cancers, several studies suggest that not all smokers, even heavy smokers develop lung cancers. Reports on none-smokers or never smokers who develop lung cancer suggest that such data overlook other more important contributing factors, particularly infective agents or pathogen-specific vaccines in the development of asthma, tuberculosis, lung and other site-specific cancers [5, 7].^10^ American health status ranks last among other healthy nations, despite the fact that USA invests the highest amount of resources for healthcare [7, 16, 20, 219, 222].

In the last six decades significant increased in soft tissue B cell-derived lymphomas (e.g., aggressive or non-aggressive forms of acute leukemia, lymphocytic leukemia, Burkett’s’ lymphoma, myelocytic leukemia, EBV-positive large B cell lymphoma in elderly, germinal center lymphoma or Kaposi sarcoma-associated herpes virus-encoded proteins in lymphoma) as well as solid tumors (e.g., liver, lung, breast, prostate, thyroid, pancreas, colon, ovarian) have been reported [5, 7, 13, 16, 20, 67, 72, 89, 101, 175, 223].

In describing cancer, every few years, cancer decision makers come up with some number and story and state that cancer is ‘too complex’; cancer is too many diseases (100 or 200 diseases). Recently, cancer was claimed to be 1000 diseases to legitimize spending funding on data sharing and aggregates!^11^ It seems that finding too many pieces of broken molecules in the cancer immune tsunami make decision makers to claim that cancer is 1000 diseases! What and how data sharing would help solving cancer problems is another puzzule! All data that are worthy (or not worthy) are published in various formats. Computational biology and data aggregation have their limits to make sense of biological activities to solve cancer problem or think about the solution. This is just another game to further postpone solving the mystery of cancer that the cancer establishment created six decades ago for maintaining control of a sick and drug-dependent society. Cancer has been made as a myth (100 or 1000 diseases) and money machine that cannot be solved. This reminds us of Phillip Zelikow *“The creation and maintenance of public myths exert a powerful influence”* [7].^12^

**Fig. 4:**
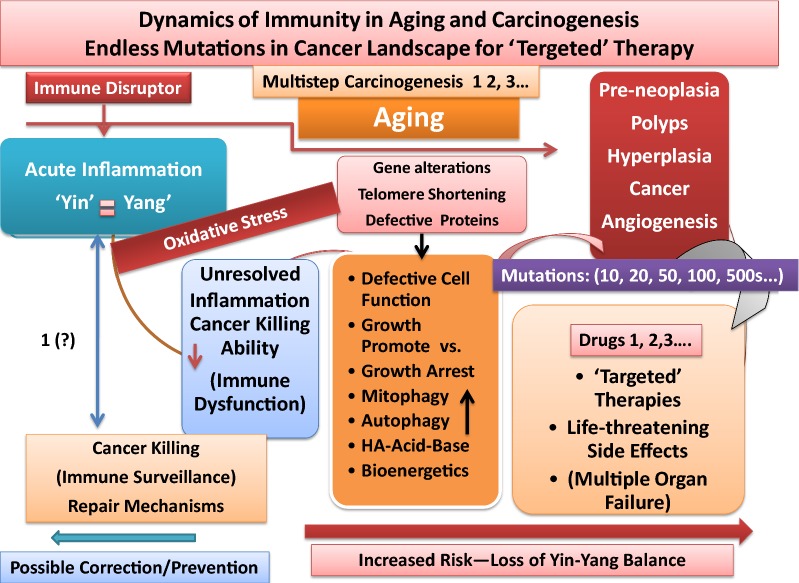
Schematic representation of complex dynamics of immunity in aging and carcinogenesis. It depicts that effective immunity or balance in Yin and Yang of acute inflammation has the ability to kill cancerous cells; that initial stages of immune dysfunction could be corrected/reversed or prevented. It depicts that unresolved inflammation and aging skew proper immune responses (immune dysfunction), associated with altered genomic stability, expression and co-expression of pro-, and anti-inflammatory factors (unresolved inflammation), alterations in mitochondrial and ribosomal functions and loss of acid–base balance in tissues that would increase risk of carcinogenesis. The complex scheme also depicts that development of cancer drugs that are based on identification of numerous mutated/defective genes or growth expression products, at late stages of cancer (within cancer molecular tsunami) are claimed as cancer ‘targeted’ therapy, ‘precision’ or ‘personalized’ medicine. Drug-induced life-threatening side effects that cause multiple organ failures (MOFs) and patients’ death are also depicted. See text

The clinical features, morphologies and pathogenicities of site-specific cancers and how to treat them, are the topics of numerous highly expensive clinical trials and basic science investigations, using modern specific technologies and models of tumors [5, 7, 16, 20, 39, 76, 79, 80, 81, 90, 92–100, 115, 116, 119–134, 182, 226–243]. However, the rates of failure in claimed ‘targeted’ drugs, ‘precision’ or ‘personalized’ medicine for solid tumors are 90% (± 5) according to governmental or private organizations [5, 7, 16, 39, 43, 79, 227–235, 237].^13^, ^14^. 15, 16 Decision makers in cancer/medical community continue to fraudulently use wrong approaches (‘molecular false flags’, based on false foundations) in cancer research and clinical trials. Endless genetic mutations have been identified in the molecular tsunami of site specific cancers for drug development that at best postpone death-sentence of patients for short durations [5, 7, 20, 45]. In such projects little regards are given to consider the serious compensatory immune mechanisms when such drugs (poisons) cause cancer relapse, cachexia, thromboembolisms, metastasis and multiple organ failures that kill patients (Fig. 4) [5–7, 16, 38, 39, 43].

A great deal of taxpayers funding are directed on detailed mechanisms of structures and substructures or potencies of tens of thousands of evolving microorganisms (viruses, bacteria, parasites) or chemical and biological carcinogens and environmentally hazardous agents, as well as endless mutated genes of growth factors or enzymes in the molecular tsunami of cancer environment. However, little/no efforts have been invested to understand what initiates pathogen-induced alterations of immune response dynamics that lead to immune tolerance, loss of mitochondrial bioenergetics (biological rhythms) and multistep carcinogenesis [5–8, 16, 39, 43, 79].

Unlike the stories that are made up by decision makers in cancer community that cancer is 100 or 1000 diseases which drag solving cancer problem, our accidental discoveries on experimental models of acute and chronic inflammatory responses demonstrated systematic developmental phases of immune dysfunction that resulted in tumorigenesis and angiogenesis. Analyses of related data and extension of these fundamental studies demonstrate that cancer is only ONE disease. Cancer is induced as the results of loss of balance in tumoricidal (Yin) vs tumorigenic (Yang) properties of effective immunity (immune tolerance) or loss of differential bioenergetics to destroy cancerous cells (Fig. 4) [5–7, 20, 39, 43].

Although recent attempts in immunotherapy seem more logical, the same reductionist views to target one or two pathways [e.g., promote dendritic cells program death ligand 1 (PDL-1) or T cell receptors] failed patients [5, 7, 20, 39, 43]. These expensive projects are not properly designed, validated or evaluated by competent and independent scientists.

Policy makers who appropriate funding for cancer research and therapy have no clue on how to assess worthiness of the tremendously expensive projects that are highly promoted by members of the cancer establishment. Such projects are considered ‘molecular false flags’, based on false foundations that destroy the precious lives of patients and drain resources to create huge corporate profits for the establishment (Fig. 4) [5, 7, 16, 20, 39, 40, 79, 95, 96, 175, 176, 226–235, 237–241].

Milton Friedman best described the situation *“If the government is put in charge of Sub Sahara, in 5* *years there will be a shortage of sands”.*^17^

## Decoy receptors-IRAK-M and cancer immune tolerance: loss of biorhythms in increased entropy and ‘Dark Energy’—toxicity to normal tissue

Recent data on computational biology or histobiological experiments by West and colleagues [78] or Pitt [208] provide insightful information that cancer entropy and higher temperature are the results of perturbations in mitosis, cell plasticity and aneuploidity in site-specific tissues. As detailed above, dysfunction of mitochondrial bioenergetics parallels loss of effectiveness in Yin (tumoricidal) and Yang (tumorigenic) properties of acute inflammation leading to polarization of immune and non-immune systems in favor of growth promotion pathways. The increased utilization of glucose by Crabtree and/or Pasteur Effects promotes disorderly growth of cancer masses, conditions that are toxic to normal cell survival [5, 55, 79, 92, 139, 185, 188, 189]. Abnormal cell division/proliferation (mitosis), aneuploidity and increased phenotype plasticity in cancer are associated with genomic instability, increased entropy and temperature, compared with surrounding tissue [5, 39, 78, 79, 208]. It is suggested that disturbance in the synchronized biological circadian rhythms of tissues could increase entropy (chaos) and temperature and create ‘dark energy’ for enhanced growth of cancer masses. Induction of ‘dark energy’ and entropy in cancer masses could draw energy from surrounding normal cells (starvation), a potentially important factor in patients’ fatigue. The use of anti-inflammatory agents (e.g., aspirin) for correcting the cancer entropy or perhaps influencing stability of chromosomal function [5, 7, 35–40, 78, 79, 208] is intriguing. Whether expression of intrinsic factors [e.g., constituent or induced receptors (PM1K, PM2K), mTOR/PI3K, IRAK-M, IL-1dRs, CAMs, PGE2, indolamine 2,3-dioxygenase, NFkB] that are anabolic during wound healing or induction of cancer growth act differently from those anti-inflammatory agents (e.g., aspirin) that are reported to improve or lower cancer entropy are among important knowledge gaps that deserve further study. The findings that NO donor molecule (*S*-nitrosoglutathione-GSNO) induces IRAK-M in LPS-activated monocytes in the presence of TNF-α are also interesting and support our definitions of tumoricidal and tumorigenic arms acute inflammation [5, 7, 35–40, 79, 89, 92, 121, 175, 193–200, 209–211].

These and related reports demonstrate elevated levels of IRAK-M in blood monocytes of patients with chronic inflammatory bowel disease or myeloid leukemia and metastasis or models of influenza also support the wound healing effects of IRAK-M [5, 39, 79, 89, 175, 191–201]. The reports that monocytes co-cultured with tumor cells or supernatant of tumor cells demonstrated significant decrease in expression of apoptotic factors such as TNF-α while increased expression of IRAK-M further support induction of immune suppression in carcinogenesis. Tumor inoculation studies of IRAK-M deficient models showed resistant to melonoma and fibrosarcoma tumor growth suggesting enhanced anti-tumor function of effector lymphocytes in the absence of IRAK-M [5, 196, 198, 213, 214]. Tumor-derived factors such as acidic gangliosides (sialic acid-containing glycosphingolipids), hyaluronan, glycosaminoglycan or C-type lectin that are generated in the extracellular matrix or plasma membrane of different cell types (e.g., chondriocytes, MΦs or DCs) are capable of stimulating expression of IRAK-M that would inhibit danger signals (e.g., TLRs) in monocytes leading to immune suppression.

The following further summarizes insights into the pathways that are involved in induction of tolerance and loss of bioenergetics in chronic diseases or cancer [5, 7, 39, 79, 89, 125–133, 139–154, 168–170, 174, 178–201]:

### Role of mTOR/PI3K, decoy receptors and IRAK-M in induction of tolerance in carcinogenesis

The mammalian or mechanistic target of rapamycin (mTOR) is a serine/threonine kinase (also known as DRAK2) and member of the phosphoinositide 3-kinase (PI3K)-family of kinases (PIKK). The super-family of mTOR/PIKK pathways is directly and indirectly involved in regulation of a wide range of tissue activities; metabolism, proliferation, differentiation, membrane lipid biosynthesis, growth and development, autophagy and immune cell responses [5, 39, 79, 178, 201, 260–267]. The two major constitutive (innate, embryonic) and induced complexes of mTORC1 and mTORC2 seem to contribute to tissue function and longevity. Molecular defects or immature biosynthesis of any members of these complex pathways have been involved in initiation of a wide range of metabolic disorders (e.g., diabetes and cardiovascular complications), infectious diseases (e.g., tuberculosis, COPD), neurological problems (e.g., autism, epilepsy, Alzheimer’s, Parkinson’s), site-specific cancers (e.g., breast, bladder, peritoneal metastasis) and other age-associated chronic illnesses. The pathways involving immune cell tolerance (immune suppression) are implicated in clinical trials such as allograft acceptance in transplanted host tissues in models of skin allograft, bone marrow or stem cell transplantation for chemotherapy-treated soft tissue sarcoma [5, 39, 79–83, 89–100, 116, 129–133, 245, 247–249]. However, mechanism of actions or usefulness of embryonic stem cell transfer for cancer therapy are debatable and yet to be understood or confirmed.

In general, growth hormones (GHs) modulate glucose uptake in insulin-dependent tissues (e.g., muscle, adipocytes). The growth hormones promoting signals involve IGF-1-independent pathways and mTORC1 complex to activate Rag-GTPase family of enzymes and lipid metabolism. Low levels of plasma lipid were suggested to promote insulin sensitivity and signaling of PI3K/AKT/mTOR [5, 7, 39, 40, 79, 193, 201–207]. Furthermore, longevity seems to be associated with altered activities of membrane-enzyme complex PI3k-AKT-mTOR pathways.

Integration of relevant data shows that PI3K/AKT is a common signaling pathway for activation of oncogenes through hypoxia, a major stimulus for expression of VEGF. Several selective inhibitors of PI3Ks (e.g., LY294002, ZSTK474, idelalisib, rituximab, SAR405, VPS34-IN1) with different effects on genetic alterations are being examined for control of inflammation in COPD, other respiratory diseases or autoimmune and neurodegenerative diseases, or for treating several cancers (e.g., CLL, non-hodgkin’s lymphoma, follicular lymphoma, breast cancer, osteoclast survival) [5, 43, 217, 218, 233, 241–245, 249]. The diverse roles of these inhibitors have primarily been shown in HIF-1α and HIF-2α and endogenous VEGF response to hypoxia and suggest that the inhibitors of different classes of PI3Ks inhibit and induce synergistically the common oncogenes, while basal hypoxia-inducible VEGF was partially inhibited.

### Tolerance in gastrointestinal (GI) tract

About 70% of body’s complex immune system is in the crucial position of digestive/gastrointestinal track. The immune composition in gut-associated lymphoid tissues (GALTs) shares some features with other tissues that are responsible for confronting and combating external harmful agents. Examples of such tissues are the skin, respiratory lung-associated lymphoid tissues (LALTs) or conjunctival-associated lymphoid tissues (CALTs) that are targets for early sensitization and tolerance (induction of Th2 phenotypes) against perennial allergens (e.g., dust mites, cat epithelium or certain environmental components) [5, 7, 8, 31, 36–41, 64, 69, 72, 79, 80, 83, 101, 106, 137, 156]. The special features of immunity and tolerization in GALTs (e.g., increased numbers of plasma cells lining of the gut epithelium for production of IgA and IgE, TLRs, enzymes and hormones) are required for maintenance of homeostasis of gut microbiota (intrinsic foreign elements) and ingested foods. In the gastrointestinal tract, tolerance against various GI bacteria (GI flora) is likely due to several regulatory/inhibitory complex molecules with IRAK-M and related immune suppressive pathways (e.g., dILRs, TNFRs or surface molecules receptors) [5–8, 36–41, 64, 69, 72, 79, 142]. Interestingly, expression of regulatory receptor molecules (IRAK-M) in epithelial lung tissue of asthmatic patients suggests induction of immune suppression also involves expression of adenosine receptors (A2A) and surface molecules of CD4^+^ T lymphocytes that could signal for mitochondrial shutdown to prevent damage to the tissue [5, 79, 142].

An overall review of numerous reports on mechanisms of tolerance or ‘intolerance’ (e.g., histamine intolerance) suggests that the initial immunity and tolerance occur during embryonic-fetus growth in lymphatic-vascular tissues, thymus, respiratory and gastrointestinal tracts under the low oxygen tension for protection of orderly growth. Fetus immunological system, studied in cord blood, has Th2 phenotypes; thus bases for protection of ‘graft-versus-host’ reactions or ‘tolerization’ [5, 69–85, 202]. After birth and during adulthood and aging process, tolerance develops toward commensal microbiota and certain endotoxins (e.g., LPS) or infective agents. Review of related data suggests that oxidative stress and aging (senescence) lead to development of tolerance (e.g., expression of IRAK-M, IL-1dRs, TNFdRs, PGE2) and/or ‘intolerance’ (e.g., increased allergic responses to innocuous or self-components) as contributing factors in skewed response network of effective immunity and induction of autoimmune or neurodegenerative diseases or cancer [5, 7, 36–40, 67, 75, 157–166, 218]. Numerous defects in cellular and membrane functions, biological components and receptor molecules [e.g., histamine, hormones (e.g., insulin-resistance, resistin), enzymes (e.g., kinases, diamine oxidase, HNMT), mutated genes, hypo-, or hypermethylated epigenetic modifications, polarized innate or adaptive immune cells and over-, or under-expressed inflammatory factors (e.g., M-CSF, IL-1dR, TNF) myeloid-derived suppressor cells, cells, impaired DNA repair pathways, autophagy or mitophagy] may be considered factors for induction of tolerance or intolerance in the development of ‘mild’, ‘moderate’ (intermediate) or ‘severe’ immune disorders including cancers [5, 39, 58, 59, 69, 84, 88, 108, 109, 116, 126, 129, 153, 154, 156–158, 164, 201–203, 215, 229, 231, 249, 250]. In the experimental models of acute and chronic ocular inflammatory diseases that we established in CALTs [5, 29, 31], whether the chronic stimulation of tissues that led to tumorigenesis and angiogenesis involved induction of tolerance by decoy receptors or IRAK-M during polarization of immune cells (e.g., TAM) are among important knowledge gaps that remain to be studied.

Nearly all age-associated chronic diseases such as metabolic disorders [e.g., type 2 diabetes mellitus (adult onset, T2-DM), cardiovascular complications, stroke] or neurodegenerative and autoimmune diseases are features of altered immunity involving polarization of immune cells and skewed expression of pro-inflammatory mediators, receptors or surface molecules (e.g., IL-6, TNFRs, M-CSF, CD11, CD34). In the case of diabetes mellitus, insulin-insensitivity are reported to increase the risk of several cancers, while it reduces risks of other cancers [5–7, 36, 202–207]. Whether accessibility of specific tissues to the released apoptotic factors cause reduced risk of specific cancers in diabetes are among questions that await future investigations. Related reports show that PI3K/AKT pathways are involved in glucose transporter-1 (GLUT-1) activities [5, 53–55, 81, 127, 175, 187–190]. Other kinases such as glycogen synthase kinases (GSK-3α, GSK-3β) play dual roles (activation and deactivation) in diverse biological activities, for growth-promoting and differentiation or growth-arresting (apoptosis), metabolism and neuronal function, embryonic development or carcinogenesis. The mechanisms of action of GSK-3 are additional examples of biorhythms or Yin–Yang of immunity, playing as tumor suppressor or tumor promoter and involving pathways of PI3K/PTEN/Akt/mTOR, Ras/Raf/MEK/ERK [5, 39, 98–101, 127, 128].

Use of diabetes drugs such as sulfonylurea and metformin seems directly influence ATP-sensitive k+ channels for enhancing membrane depolarization of pancreatic beta cells and stimulating exocytosis of insulin granules [5, 54, 89, 180, 202–207, 253]. The suggested mechanisms and clinical values or efficacy and safety of such agents in diabetes are controversial. In general, these agents seem to support that cellular exocytosis is energy-dependent processes in immune and non-immune cells/tissues. Diabetes (hyperglycemia) and related metabolic disorders are considered immune disorders that initially influence the metabolic pathways for glucose transport and metabolism. Impaired glucose transport and utilization in these metabolic disorders, are associated with induction of Il-6, T cell activation and generation of memory or regulatory cells (Treg), pathways that require additional sources of energy from fatty acid oxidation for glycolysis and glutaminolysis, as alternative or compensatory mechanisms for impaired mitochondrial oxidative phosphorylation. Under these conditions, cell surface ligation and activation of membrane phospholipases (e.g., PLC) or perhaps metabolism of arachidonic acid (AA) and activation of cyclooxygenase/lipooxygenase pathways, as well as, release of low level histamine, would allow mobilization of intracellular Ca^2+^ under impaired ER and T cell-dependent plasma membrane influx of Ca^+2^ and other ion channels [e.g., calcium release-activated channels (CRAC), H^+^/Ca^2+^/K^+^ or Na^+^ exchangers] [5, 39, 47, 81, 140–143, 179, 180, 184, 203, 239, 253]. Hyperglycemia of diabetes could differentially interfere with transport and metabolism of nutrients, amino acids or solutes/osmolytes, (e.g., vitamin C, pyridoxine/pyridoxal phosphate, myo-inositol, leu, ala, gly) in tissues that are insulin-dependent or insulin-independent for glucose transport and metabolism and could change extra-, intracellular structures (e.g., protein/lipid glycosylation, basement membrane collagen synthesis) [5, 7, 36, 39, 79, 203–207, 221, 250–260]. Related data on obesity show low-grade inflammation and impairment of insulin receptor signaling and insulin resistance are mediated through the complex and interdependent stress kinases [e.g., p38, mitogen-activated protein kinase (MAPK), c-Jun NH2-terminal kinase (inhibitor of NF-kB kinase-β-IKKβ), AMP-activated protein kinase, protein kinase C, Rho-associated coiled-coil containing protein kinase, RNA-activated protein kinase] to phosphorylate the key regulators of glucose homeostasis in various tissues. The phosphorylation of serine residues of insulin receptors (e.g., IRS-1) results in diminished enzymatic activity of PI3K/Akt pathway, important mechanisms that contribute to insulin resistance in type 2 diabetes mellitus or induction of cancer growth [5, 36, 52–54, 102, 178, 203–207, 221]. Review of a number of reports also suggests that insulin-insensitivity and hyperglycemia (glucose toxicity and high circulating glucose levels) alter mTOR complexes (mTORC1, mTORC2) and mediate several interdependent pathways of metabolism, ribosomal biogenesis and autophagy. Whether caloric restriction (CR) and increased insulin sensitivity via decreased signaling in mTOR pathways, promotes endocrine factors and lifespan are subjects of recent debates [5, 72, 79, 202–207].

Therefore, it is logical to consider that PI3K/AKT/mTOR activities play crucial and interdependent roles in contributing to the induction of tolerance, growth and metabolism of tissues that would influence innate/intrinsic pathways under hypoxic conditions and mitochondrial dysfunction.

### Biology of IRAK-M and soluble hormones in immune tolerance: violations of biological circadian rhythms in carcinogenesis

Effective immunity requires elaborate and precise communication with a variety of hormones [e.g., estrogen, α-melanocyte-stimulating hormone (MSH), insulin, TSH, cortisol, adiponectin] that are induced or secreted from tissues and organs (e.g., thyroid, neuro-endocrine, liver, adrenal glands, adipocytes, breast, ovary, thymus) for routine maintenance of health throughout life [5, 7, 36, 39, 40, 71, 72, 202–207]. These hormones have anti-inflammatory properties and are often involved in wound healing (Yang) events. The actions of these hormones are regulated by a number of receptor molecules and inhibitors and their role fluctuate at different stages of life [5, 7, 39, 74, 75, 262]. For example, review of several elegant reports demonstrate that growth promoting factors such as adiponectin, insulin or anti-infective mediators that induce IRAK-M expression require activation of phosphatidyl inositol 3-kinase (PI3K), protein kinase B (AKT/PKB/mTOR) or ERK pathways to induce macrophage endotoxin tolerance and signal for wound healing and immune suppression [5, 7, 36, 39, 178–182, 195–207].

## Induction of ‘Dark Energy’ in cancer mitosis and proliferation: Fatigue syndrome, a working hypothesis

As noted above, continued proton pumping and generation of electricity are required for numerous routine cellular activities such as transport of ion/solute and metabolites, lysosomal digestion and protein recycling, degradation of pathogens’ structural proteins-lipids-genes for immune recognition, activation and cellular proliferation. Proper functioning of mitochondria as energy power plant is crucial for proton pumping during routine metabolism or fighting against harmful elements (intrinsic and extrinsic). Mitochondrial function depends on presence and function of essential basic building blocks (e.g., pyruvate-shuttle, pyruvate carrier proteins, TCA cycle mediators) to generate energy and maintain crosstalk between cytoplasm and mitochondria, at moment notice for production of high energy and oxidants to fight pathogens (Yin) and also during downtime (Yang) for biosynthesis of intermediates of TCA cycle and preservation of energy.

**Fig. 5:**
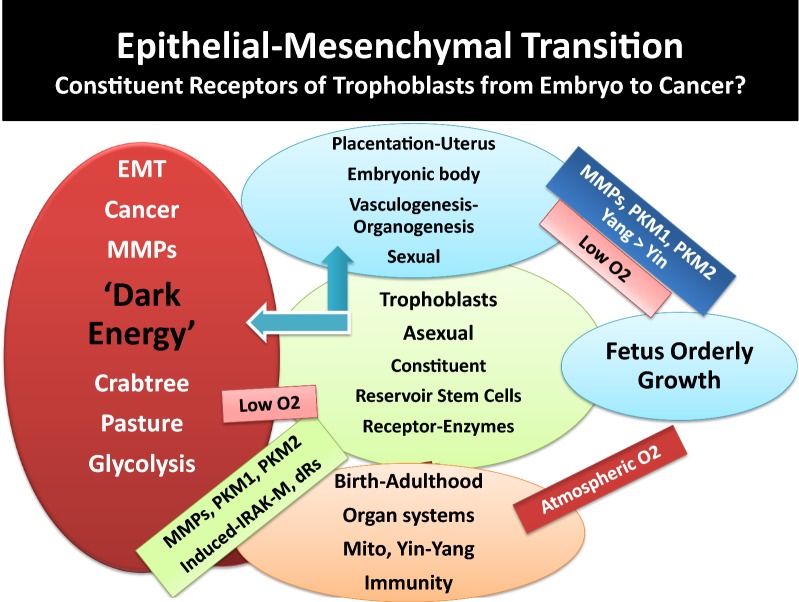
Schematic representation of loss of biorhythms, expression of constituent receptors and induction of tolerance for epithelial–mesenchymal transition (EMT) during cancer proliferation and growth. The scheme represents activation of trophoblasts growth factors that are required for placentation during embryonic and orderly fetus growth (constituent) and receptors including pyruvate kinases receptors and hormones. Induction of anabolic factors increase lawless growth of cancer cells and increase metabolism through glycolysis under mitochondrial dysfunction and hypoxia, features resembling/mimicking aspects of orderly growth of fetus. Expression of embryonic growth factors facilitate increased glucose metabolism through glycolysis, Pasteur and Crabtree effects and induction of immune tolerance creating ‘dark energy’ that enhance entropy, mitosis and proliferation of cancer masses, conditions that are toxic for normal cell survival. See text

Loss of synchronized network of Yin and Yang of effective immunity is intimately entangled with energy drain and electronic charges of important proteins for integrity of intra-, extra-cellular membrane structures. It is suggested that the altered (increased) entropy or temperature creates ‘dark energy’ around cancer masses and drain energy from neighboring normal tissues. The differential bioenergetics that are required for maintenance of circadian rhythms in health could be shifted/switched under oxidative stress, during the induction of tolerance and mitochondrial shutdown in exchange for increased glycolysis, enhanced growth of cancer cells and associated increase in entropy and temperature. The shifts in energy distribution and enhanced entropy around cancerous cells (‘dark energy’) could alter energy requiring events of protein electrical charges (e.g., negative charges, H-bonds, hydrophobic/hydrophilic ratios) for proper cellular functions. It is further suggested that impaired electrical charges could adversely influence protein folding that would alter a number of cellular activities (e.g., water, nutrient and ions channels, transporters, structural proteins). Cumulatively, altered energy-requiring biological activities could limit energy utilization in normal neighboring cells, a potential factor in symptom of fatigue observed in cancer patients. Enhanced ‘dark energy’ and entropy create asymmetry that could force the normal cells from oscillating at required energy (loss of biorhythms) for cell survival (Fig. 5) (manuscript in preparation). The extent of progressive complex interactions between growth of tumor cells and host environment could dictate and direct clinical response outcomes. Potential influence of oxidative stress in shifting the ratios of innate/adaptive production of mTORC1/mTORC2 during induction of tolerance and activation of Crabtree and/or Pasteur Effects in creating differential entropy and ‘dark energy’ and disorderly growth of cancer masses are important topics that require systematic studies for effective control of cancer cell growth.

### Summary and future directions

*While falsely*-*based science will not hide the scientific truth at the end, it creates confusion and chaos that benefit those who gain control of public health at high cost to society.*

In this comprehensive article, the author presented sufficient evidence to suggest that cancer is an induced disease of Twentieth century, facilitated by the decision makers in cancer/medical establishment when the public was immunized by virus-contaminated polio vaccines in 1955s, despite extensive evidence that viruses cause cancer. It was demonstrated that pathogen-specific vaccines and ingredients weaken/retard immunity, not promote it. Cancer was shown to be the symptom of the loss of differential bioenergetics of effective immunity (Yin–Yang) that is responsible for fighting and destroying cancerous cells or any other intrinsic and extrinsic hazardous materials. Under immune suppression cancerous cells slowly or aggressively take over the machinery of host for its enhanced and lawless growth, under hypoxic conditions and mitochondrial dysfunction. The energy for abnormal growth of cancerous cells is supplied by increased glucose uptake and altered tissue metabolism via glycolysis and Crabtree and Pasteur Effects that create ‘dark energy’ and entropy, conditions that are toxic to normal cells. Induction of ‘dark energy’ is characteristics of disorderly growth and proliferation of cancer cells that drain the energy of neighboring normal cells, potentially contributing to the observed fatigues in patients.

It was hypothesized that mitochondria and Yin and Yang of effective immunity and biological reprogramming develop after birth when newborn is exposed to atmospheric pressure and environmental conditions. Longevity and unresolved inflammation were defined as alterations in immune and non-immune response dynamics. Tardiness of immunity (loss of balance in Yin and Yang) leads to induction of ‘mild’, ‘moderate’ or ‘severe’ immune disorders, conditions that are associated with altered mitochondrial function. Insufficient circadian rhythms (skewed biological clocks) were suggested to be the results of one or more mutations or deficiencies in the circadian clock genes that influence the synchronized communications among biological oscillators (positive and negative rhythms) or ‘effective acquisition time’ of immunity. Lawless growth of cancer cells is peculiarly comparable to the orderly (one way) growth of fetus that involve activation of trophoblasts factors and constituent receptors that are required for fetus orderly growth under hypoxic conditions (Fig. 5).

The current reductionist approaches to cancer science and therapy are conducting numerous out-of-focus and fuzzy projects projects on identification of one or combination of mutated genes or expression products using expensive and specific technologies as bases for drug development. The outcome failure rates of such reductionist, fraud and chaotic projects are 90% (± 5) that destroyed the precious lives of millions, but generated huge corporate profits for the cancer/medical establishment (e.g., government, Big pharma, organizations and largest lobbying group and ‘philanthropists’/businessmen who support medical education programs) [5, 7, 20, 39, 43]. Again, targeting young and old population to be vaccinated by pathogen-specific vaccines (e.g., HPV, flu, measles, meningitis) weaken (not promote) immunity. The pathogen-specific vaccines and ingredients are viewed as ‘antigen overload’ overwhelming or skewing immunity to clear and resolve immune/inflammatory responses. Tardiness of immune responses is the bases for induction of a wide range of health problems such as asthma, autoimmune and neurodegenerative diseases or cancers in old and young populations.

After investing several trillions of dollars of taxpayers’ and private organizations funding for cancer research and treatment, the decision makers have yet to seriously consider the need for understanding the following scientific common senses and logics:

- What are the early changes in complex network of immune responses that lead to genetic instability, loss of biorhythms, mitochondrial dysfunction and altered metabolism that lead to immune tolerance toward multistep carcinogenesis and angiogenesis.
- How to develop universal vaccines and prophylactic agents that promote body’s natural immunity for maintaining the autonomous, sympathetic and parasympathetic or Yin and Yang, biological circadian rhythms that are required for improving public health and preventing majority of chronic diseases or cancer.

Future research directions require focusing on systematic and logical studies that are outlined in the following overall topics [5, 7, 20, 39, 72, 175]:

i. Time-course kinetics of immune response dynamics are fundamental and essential first steps in understanding details of host interactions with stimuli (infective agents, environmental and biological hazards). The extent of damage that specific immune disruptors impose on bioenergetics and architectural integrity and function of affected tissues during developmental phases of multistep disease processes are among important topics that deserve detailed studies. Identification of shared or special features of early events during host–pathogen interactions that alter immune response profiles are among important knowledge gaps that require detailed studies.
ii. Potential reversibility of early stages of inflammation-induced immune dysfunction including alterations in cellular chromosomal/genetic material that would lead to cellular growth promotion and genesis of hyperplasia, neoplasia/pre-cancer or cancer-malignancy deserve detailed studies.
iii. The quality (nature) and quantity of initial acute immune responses (strong or weak) and the generation of histamine and other pro-, or post-inflammatory mediators could influence subsequent responses such as the extent of rejection or penetration of antigen in subepithelial tissue, vascular hyperpermeability and tissue integrity during Yin and Yang activities and clearance of antigen.
iv. It is important to understand the influence of mitochondrial dysfunction and energy shifts during increased glycolytic pathways that result in adaptation of cancer cell growth and immune tolerance. Induction of tolerance could change mitochondrial-dependent activities of cMyc, Alt/PTEN or p53 and related pathways and alter apoptosis (Yin) events leading to proliferation and entropy or ‘dark energy’ in cancer mass.
v. Understanding the thermodynamics and biological communications of high energy-consuming and voltage-dependent pathways in apoptosis (Yin, tumoricidal) that are involved in destroying defective cancer cells are important topics that are not fully understood.
vi. Stimuli-induced redox-sensitive mitochondrial transition pore (MTP) that opens to the cytoplasm, followed by depolarization, electron flux in the electron transport chain (ETC) during production of electron donors (NADH and FADH2) that would increase the level of reactive oxygen species (ROS) are crucial topics that require better systematic studies.

Minor or major heterogeneities in intrinsic biology and genetic makeup of individuals often lead to heterogeneities in response profiles toward different biological insults (immune disruptors). These confounding factors present unique challenges and opportunities to overcome in future research. However, the outcomes are expected to be rewarding as the truth in science has always advanced us to extraordinary achievements in many biomedical fields. Solving cancer problem is not an exception if the business of cancer did not overwhelm the search for scientific facts and logics.

## Concluding remarks

For over six decades the reductionist approaches of decision makers in cancer science popularized the notion that cancer is too many diseases (100, 200 or 1000); cancer is too complex to solve; that they have made ‘extraordinary advances’ and need more money to further progress on the war against cancer. The decision makers now make up other stories that they need to aggregate and share data and require more resources and time to solve cancer mystery. The truth about claims of ‘targeted’ therapy based on identification of endless defective molecular entities is that such mindless and chaotic approaches failed patients at the rate of 90% (±5), costing the loss of millions of precious lives and financial toxicity to the society. All relevant data are available in various medical and basic science disciplines. Integration of quality data on how cancer is initiated has been practically ignored and its systematic investigations are not allowed when competent professionals propose more logical and cost-effective studies to help understand cancer biology and how to prevent or treat it. As author presented in the last couple of decades, analyses of data from her accidental discoveries on experimental models of ocular inflammatory diseases and extension of relevant data on multidisciplinary fields of medical and immunological sciences suggest that cancer is only one disease. Disorderly growth of cancer cells is the results of loss of autonomic and synchronized balance in tumoricidal (Yin) and tumorigenic (Yang) properties of immunity to arrest cancer cells. Loss of differential bioenergetics in Yin (high energy, tumoricidal) and Yang (low energy, tumorigenic) pathways often leads to ‘mild’, ‘moderate’ or ‘severe’ immune disorders or cancer.

The goal of this comprehensive perspective was to extend the design of a roadmap as comprehensive as possible, by analyzing relevant data on major biological features at different stages of life. In the process of integrating and connecting the informational dots, important knowledge gaps that are worthy of future investigation were revealed. Integrating and disseminating relevant information are important for broadening the scope of intellectual understanding on the complex dynamics of effective immunity that lead to effective promotion and maintenance of health.

After all ‘*we may be intelligent, but if not able to think and love well being of others, we use the intelligence against humanity’* [?].

## Abbreviations

AA: arachidonic acid
ASPH: aspartyl-asparginyl β-hydroxylase
CALTs: conjunctival-associated lymphoid tissues
CAM: cell adhesion molecule
CRP: C-reactive protein
DAMP: damage-associated molecular pattern
ECM: extracellular matrix
GSK-3α, GSK-3β: glycogen synthase kinases
GALTs: gut-associated lymphoid tissues
ICAM: intracellular CAM
IRAK-M: interleukin-like activated kinase M
HMGB1: high-mobility group box 1
HPV: human papilloma virus
LALTs: lung-associated lymphoid tissues
LPS: lipopolysaccharide
LTA: lipoteichoic acid
mTOR: mammalian/mechanistic target of rapamycin
MAPKs: mitogen-activated protein kinases
MCP-1: monocyte chemoattractant protein 1
MOFs: multiple organ failures
NETs: neutrophil extracellular traps
PGN: peptidoglycan
PI3K: phosphoinositide (phosphatidyl inositol) 3-kinase
PIKK: PI3K kinase
PRRs: pattern recognition receptors
PEP: phosphoenol pyruvate
ROC: Receptor-operated channel
SAPK: stress-activated protein kinase
TLRs: toll-like receptors
TRPM4: transient receptor potential cation channel, subfamily 4
TCA: tri-carboxylic acid
TNF: tumor necrosis factor
TNFRs: TNF Receptors
VCAM: vascular CAM

## Footnotes

1 Cancer/Medical establishment is referred to the most powerful decision making group within the Government (NIH/NCI-FDA-CDC-DHHS), Big pharma, major organizations (AACR, ACS, ASCO, AMA), cancer research and treatment centers, major medical schools. Members of establishment (5–10 × 10^3^) with carrier longevity of 35–65 years are decision makers in charge of research directions, publicity and funding. The temporary or permanent handlers of establishment (directors of institutes, divisions or departments, deputies and staff (30–100 × 10^3^) and the world’s largest lobbying group (3–5 × 10^6^) carry out the directions of members of establishment. War on cancer has been made as an imaginary problem that cannot be solved for over six decades [5, 7, 16].

2 Health Impact News December 10, 2017—reported from World Mercury Project Team—Anthrax Vaccine: Safety Concerns-‘Pilots Would Rather Leave the Military than be Forced to get Anthrax Vaccine’.

3 US Senator Grassley Investigates Vaccine Corruption in Unsanctioned Experimental Herpes Vaccine: Human Research. Health Impact News, January 12, 2018.

4 Wagstaff A, Culyer AJ. Four decades of health economics through a bibliometric lens. J Health Econ. 2012;31:406–39. 10.1016/j.jhealeco.2012.03.002.

5 Khatami M (2016) Book review: Safety concerns for HPV vaccination of young generation, paid by Obamacare and V.P. Biden Moonshot initiative. Global Vaccines and Immunology, ISSN: 2397-575X, 2016 https://oatext.com/pdf/GVI-1-118.pdf.

6 Tom Valentine: Polio vaccine spreads cancer. Dark Politricks. Part II, SHOAH, June 21, 2011.

7 Brian Shilhavy. The Truth About Vaccines: DocuSeries with 60 Top Health Experts Reveal What is Censored in Mainstream Media, Jan 13, 2018; U.S. Government continues to pay out millions to victims injured by the flu shot. Reflects report from Department of Justice (DOJ) on cases settled for vaccine injuries and death mandated by National Vaccine Injury Compensation Program (NVICP), March 8, 2018.

8 Daily Mail-UK Media Publication.”HPV vaccine destroying the lives of ‘Thousands’ of girls”, Accessed from Health Impact News, Sept. 28, 2017.

9 Gordon Duff. Is American medicine a war crime? Veterans Today, July 3, 2012. http://www.veteranstoday.com/2012/07/03/is-american-medicine-a-war-crime/.

10 Holtzman A: Dr. Leon Dmochowski and Dr. R. Lee Clark—Truth Tobacco Industry Documents.5-page Letter, to Paul D. Smith, VP and General Counsel, Philip Morris Inc., January 06, 1965. https://industrydocuments.library.ucsf.edu/tobacco/docs/[ID], Ness Motley Law Firm. https://industrydocuments.library.ucsf.edu/tobacco/docs/qymd0040—Bates Number: 10051001761005100180 [7].

11 The Cancer letter, “His six-month ‘listening tour’ almost over, Sharpless discusses his vision for NCI”, February 23, 2018.

12 Phillip Zelikow was one of the architects behind creation of war against powerless nations.

13 In February 2012, National Cancer Institute (NCI) Board meeting (NCAB) report on cancer therapy, it was admitted that success rate being 15%; in March 2012, during Metabolon conference in Bethesda, Maryland, company professionals reported that 95% of cancer drugs that are developed fail [7].

14 June 1, 2012, E Berger (CNBC program) in an interview with then president of MD Anderson, DePinho confirmed that 95% of cancer drugs for solid tumors fail—http://blog.chron.com/sciguy/2012/06/m-d-anderson-president-goes-on-cnbc-extols-his-own-company.

15 Brill S. (2013) Special Report, Bitter pill: Why medical bills are killing us. Time (USA edition) March 4, 2013, 16-55.

16 Beil L. (2012) How much would you pay for 3 more months of life? Newsweek, 9/3/2012. Vol. 160, Issue 10, 40-44.

17 Milton Friedman, Nobel Laureate in Economic Sciences (1976), for his achievements in the field of consumption analyses, monetary history and theory and demonstration of the complexity of stabilization policy. Source: The Nobel Foundation.
